# Rhodopsin optogenetic toolbox v2.0 for light-sensitive excitation and inhibition in *Caenorhabditis elegans*

**DOI:** 10.1371/journal.pone.0191802

**Published:** 2018-02-01

**Authors:** Amelie Bergs, Christian Schultheis, Elisabeth Fischer, Satoshi P. Tsunoda, Karen Erbguth, Steven J. Husson, Elena Govorunova, John L. Spudich, Georg Nagel, Alexander Gottschalk, Jana F. Liewald

**Affiliations:** 1 Buchmann Institute for Molecular Life Sciences, Goethe-University, Frankfurt, Germany; 2 Department of Biochemistry, Chemistry and Pharmacy, Institute of Biophysical Chemistry, Goethe-University, Frankfurt, Germany; 3 International Max Planck Research School in Structure and Function of Biological Membranes, Frankfurt, Germany; 4 Systemic Physiological & Ecotoxicological Research (SPHERE), University of Antwerp, Antwerp, Belgium; 5 Center for Membrane Biology, Department of Biochemistry and Molecular Biology, University of Texas Health Science Center at Houston, McGovern Medical School, Houston, Texas, United States of America; 6 Department of Biology, Institute for Molecular Plant Physiology and Biophysics, Julius-Maximilians-University of Würzburg, Würzburg, Germany; 7 Cluster of Excellence Frankfurt - Macromolecular Complexes (CEF-MC), Goethe University, Frankfurt, Germany; Indiana University School of Medicine, UNITED STATES

## Abstract

In optogenetics, rhodopsins were established as light-driven tools to manipulate neuronal activity. However, during long-term photostimulation using channelrhodopsin (ChR), desensitization can reduce effects. Furthermore, requirement for continuous presence of the chromophore all-*trans* retinal (ATR) in model systems lacking sufficient endogenous concentrations limits its applicability. We tested known, and engineered and characterized new variants of de- and hyperpolarizing rhodopsins in *Caenorhabditis elegans*. ChR2 variants combined previously described point mutations that may synergize to enable prolonged stimulation. Following brief light pulses ChR2(C128S;H134R) induced muscle activation for minutes or even for hours (‘Quint’: ChR2(C128S;L132C;H134R;D156A;T159C)), thus featuring longer open state lifetime than previously described variants. Furthermore, stability after ATR removal was increased compared to the step-function opsin ChR2(C128S). The double mutants C128S;H134R and H134R;D156C enabled increased effects during repetitive stimulation. We also tested new hyperpolarizers (ACR1, ACR2, ACR1(C102A), ZipACR). Particularly ACR1 and ACR2 showed strong effects in behavioral assays and very large currents with fast kinetics. In sum, we introduce highly light-sensitive optogenetic tools, bypassing previous shortcomings, and thus constituting new tools that feature high effectiveness and fast kinetics, allowing better repetitive stimulation or investigating prolonged neuronal activity states in *C*. *elegans* and, possibly, other systems.

## Introduction

Optogenetics allows versatile modulation of the activity of cells with high spatiotemporal resolution using light [1, 2]. Many optogenetic tools have been developed in the past decade. Most numerous are the microbial opsins, which mediate ionic currents across the plasma membrane [3]. Following heterologous expression, they can be used to gain control over excitable cells in living tissue or freely behaving animals, and even patterns of neuronal activity can be mimicked [4]. Rhodopsin optogenetic tools are used in various model organisms, from the nematode *C*. *elegans* [5–9] to non-human primates like the macaque [10, 11], to study basic mechanisms of synaptic transmission but also the function of neuronal circuits generating complex behaviors.

A range of optogenetic tools is available for activation–i.e. depolarization (e.g. ChR2)—or inhibition—i.e. hyperpolarization (e.g. NpHR) of excitable cells [12–21]. Nevertheless, current tools are still limited in their applicability, mostly as temporal control (e.g. repetitive or long-term excitation) or effectiveness are often not sufficient for more complex protocols, particularly in invertebrate models where ATR supplementation is required. Thus, there is a continuous interest to expand the toolbox in order to allow for a broader range of precise applications. Novel rhodopsins are either identified by screening sequenced genomes or by site-directed mutagenesis of established proteins [17, 22–26]. In case of ChR2, point mutations were identified that affect its membrane expression, ion selectivity, conductivity, kinetics, or action spectrum [27, 28]. The H134R mutation increases plasma membrane expression [29] as well as steady-state photocurrents, and data from *C*. *elegans* and other expression hosts suggest that H134R stabilizes incorporation of all-*trans* retinal (ATR) [7, 8, 27, 30]. ChR2(T159C) exhibits improved plasma membrane expression and increased channel conductivity, leading to larger photocurrents [21, 31]. The CatCh (calcium translocating channelrhodopsin; L132C) variant increases Ca^2+^ conductivity and apparent light sensitivity, presumably due to charge effects of Ca^2+^ at the cytosolic leaflet of the plasma membrane improving activation of voltage-gated Na^+^ channels [32]. Thus, even very low light intensities can trigger action potentials.

Other ChR2 mutants affect photocycle kinetics and are useful for particularly fast or long-lasting depolarization. ChETA (E123T) accelerates channel closing and recovery from the desensitized state and can trigger exceptionally fast depolarizations, allowing elicitation of action potentials with high frequency (up to 200 Hz) [33]. Likewise, Chronos and Chrimson enable fast spiking [19]. In contrast, mutation of ChR2-Cys128 to Thr, Ala, or Ser, or of Asp156 to Cys were shown to decelerate closing kinetics [8, 34, 35]. Following brief photoactivation, the open channel is stabilized, allowing depolarizations from seconds to minutes. Consequently, the operational light sensitivity is increased. Subsequent inactivation using a different wavelength enables a step-like switching. This ‘step-function’ property enables to mimick ongoing neuronal activity or reducing action potential thresholds. Yet, several properties limit the utility of such ‘slow’ variants for long-term depolarization in the range of hours, as could be of interest in developmental biology [8]. First, with closing kinetics in the range of seconds to minutes, they require repetitive stimulation to induce sustained depolarization for longer time periods. However, ChR2(C128S) and ChR2(C128T) enter long-lived desensitized states after channel closing, impeding immediate reactivation [34]. Furthermore, ChR2(C128S) requires continuous supplementation of ATR to maintain sustained excitability [8]. The mutation may lead to loss of ATR, as indicated in resonance Raman spectroscopy measurements: The P353 photo-intermediate exhibited a signature of hydrolysis and reformation of the Schiff base, and transient formation of free ATR [30]. This affects ChR2(C128S) utility in organisms that require addition of ATR, like *C*. *elegans*, *Drosophila*, and to some extent, *Xenopus* oocytes. Thus, ChR2 variants with further delayed closing kinetics combined with enhanced off-ATR excitability would be highly desirable.

Optically triggered hyperpolarization, leading to transient inactivation of excitable cells, is a potent means to gather information on the function of specific neurons. Commonly, light-driven ion pumps (e.g. NpHR) are agents of choice for membrane hyperpolarization [6, 15, 16, 18]. However, the active transport of pumps restricts the utility of these tools. At least under certain conditions, e.g. at high depolarization where the driving force for Cl^-^ influx is high, ion channels would yield much higher current. Since only one ion is moved per photocycle, pumps require continuous stimulation, and they cannot take advantage of photocycle-affecting mutations [16]. Yet, as their action is usually independent of the membrane potential, pumps are also advantageous over channels, particularly at hyperpolarized potentials.

In search for a light-gated Cl^-^-channel ChR2 was modified by site-directed mutagenesis: iC1C2, a ninefold mutated version of the C1C2 chimera [26], or ChloC (Chloride-conducting ChR2 [17]). As the latter retained some proton conductance, causing small, yet significant depolarization, two additional amino acids in the proton pathway were substituted, generating improved ChloC (iChloC) [36]. iC1C2 was improved to iC++, with 15x higher currents [37]. Even more effective are the natural anion channel rhodopsins which were identified in cryptophyte algae (ACR1, ACR2; here used for *Gt*ACR1 and *Gt*ACR2 from *Guillardia theta*)[24], and in additional species a whole range of channels was analyzed [38]. Their peak currents are larger than those of iChloC, iC1C2 or iC++ (ACR2 conducts ≈3-fold higher current than iC++ [24, 37]). While ACR2 exhibits particularily fast kinetics, ACR1 shows larger plateau currents and has a slightly red-shifted action spectrum (515 nm peak compared to 470 nm for ACR1). Being fast, exhibiting a ≈1000-fold higher operational light sensitivity (ACR2; Ref. [24]) than previously used hyperpolarizers and showing a very high conductance, ACRs are particularily interesting for optogenetic inhibition, as was recently demonstrated in *Drosophila* [39].

In a screening study for ACR homologues, ZipACR was identified as another promising tool for optogenetic silencing. In HEK293 cells, it induced even larger currents than ACR1, and featured an unprecedentedly fast conductance cycle. In cultured mouse hippocampal neurons, it enabled inhibition of individual spikes up to 50 Hz [38]. Searching for tools with slow closing kinetics, Cys102 in ACR1 was identified as the corresponding residue to Cys128 in ChR2. Accordingly, mutation in ACR1(C102A) resulted in decreased current amplitudes but also in dramatically prolonged current decay, making it 100-fold slower than wild-type ACR1 [40, 41].

In the present study, we explored the use of novel de- and hyperpolarizing optogenetic tools in *C*. *elegans*, with a focus on the utility in this important model system in behavioral, cellular and molecular neurosciences [13, 42–45]. Because of its small, well-defined nervous system [46] and its transparency, it is ideally suited for optogenetics. Tools affecting membrane potential can be tested in body-wall muscles (or cholinergic neurons), causing muscle contraction or relaxation (measured by video microscopy), and electrophysiologically accessible currents. For depolarizers, we combined several single mutations already known for ChR2 [7, 8, 21, 27, 29, 31, 32, 35], aiming to generate variants that unify favorable attributes of these mutations, particularly for long-term depolarization. Additionally, we examined some known variants that had not previously been tested in *C*. *elegans*. We analyzed the extent, duration, and repeatability of activation, operational light-sensitivity, and excitability after removing the animals from of ATR. The very efficient quintuple mutant ‘Quint’ allows depolarization for 8–10 h following a single light pulse, while ChR2(H134R;D156C) exhibited highest operational light sensitivity. For hyperpolarizers, we probed the potential of ACR1, ACR2, ACR1(C102A), and ZipACR as alternatives to the commonly used light-driven pumps. Particularily ACR1 and ACR2 led to stable effects with fast kinetics and (at depolarized membrane potentials) higher photocurrents compared to NpHR.

## Materials and methods

### *C*. *elegans* culture and transgenic animals

Nematodes were cultivated on NGM plates seeded with *E*. *coli* OP-50 bacteria, optionally supplemented with ATR (Sigma-Aldrich, Germany) [13]. Transgenic animals were obtained by microinjection using varying concentrations of plasmid DNA. Strains ZX1788, ZX2022, ZX2023, ZX2024, ZX2026, ZX2206, and ZX2207 were generated via injection of 30ng/μl plasmid DNA each, combined with 5ng/μl of the co-marker pmyo-3::mCherry (ZX2024, ZX2026), 10ng/μl of pmyo-2::mCherry (ZX2022, ZX2023, ZX2206, ZX2207), or 10ng/μl of pelt-2::mCherry (ZX1788) into gonads of N2 by standard procedures [47]. The reason for using different DNA amounts was that some constructs did not yield healthy strains when expressed at higher levels or that different amounts of plasma membrane expression were achieved. Since it is virtually impossible to control the precise amount of plasma membrane content of heterologously expressed membrane proteins, we rather generated several transgenic lines for each construct, choosing lines with similar transgene transmittance, mosaicism and membrane expression. Strains expressing pmyo-3::ChR2(C128S;H134R)::YFP and pmyo-3::ChR2-Quint::YFP were produced by injection of 100ng/μl (ZX1295, ZX1299), 40ng/μl (ZX1296, ZX1300), 10ng/μl (ZX1297, ZX1301), or 2ng/μl (ZX1298, ZX1302) plasmid DNA. CatCh-related animals were obtained by microinjection of 80ng/μl plasmid DNA combined with 1.5ng/μl of pmyo-2::mCherry (ZX1826, ZX1827, ZX1830).

The following transgenic strains were used or generated: **ZX299**: *lin-15(n765ts-);zxEx22[pmyo-3*::*ChR2(H134R)*::*YFP;lin-15+]*, **ZX444**: *lin-15(n765ts-);zxEx301[pmyo-3*::*NpHR*::*ecfp;lin-15+]*, **ZX838**: *lin-15(n765ts-);zxEx423[pmyo-3*::*ChR2(C128S)*::*YFP;lin-15+]*, **ZX954**: *lin-15(n765ts-);zxEx468[pmyo-3*::*ChR2(C128S;H134R)*::*YFP;lin-15+]*, **ZX1166**: N2;*zxEx536[pmyo-3*::*ChR2(T159C)*::*YFP;pmyo-2*::*mCherry]*, **ZX1167**: N2;*zxEx537[pmyo-3*::*ChR2(H134R;T159C)*::*YFP;pmyo-2*::*mCherry]*, **ZX1295**: *lin-15(n765ts-);zxEx477[pmyo-3*::*ChR2(C128S;H134R)*::*YFP(100ng/μl);lin-15+]*, **ZX1296**: *lin-15(n765ts-);zxEx478[pmyo-3*::*ChR2(C128S;H134R)*::*YFP(40ng/μl);lin-15+]*, **ZX1297**: *lin-15(n765ts-);zxEx479[pmyo-3*::*ChR2(C128S;H134R)*::*YFP(10ng/μl);lin-15+]*, **ZX1298**: *lin-15(n765ts-);zxEx480[pmyo-3*::*ChR2(C128S;H134R)*::*YFP(2ng/μl);lin-15+]*, **ZX1299**: *lin-15(n765ts-);zxEx477[pmyo-3*::*ChR2-Quint*::*YFP(100ng/μl);lin-15+]*, **ZX1300**: *lin-15(n765ts-);zxEx478[pmyo-3*::*ChR2-Quint*::*YFP(40ng/μl); lin-15+]*, **ZX1301**: *lin-15(n765ts-);zxEx479[pmyo-3*::*ChR2-Quint*::*YFP(10ng/μl);lin-15+]*, **ZX1302**: *lin-15(n765ts-);zxEx480[pmyo-3*::*ChR2-Quint*::*YFP(2ng/μl);lin-15+]*, **ZX1788**: N2;*zxEx1036[pmyo-3*::*ChR2(H134R;D156C)*::*YFP(30ng/μl);pELT-2*::*mCherry]*, **ZX1826**: N2;*zxEx740[pmyo-3*::*ChR2(C128S;L132C;H134R)*::*YFP;pmyo-2*::*mCherry]*, **ZX1827**: N2;*zxEx741[pmyo-3*::*ChR2(L132C)*::*YFP(80ng/μl);pmyo-2*::*mCherry]*, **ZX1830**: N2;*zxEx744[pmyo-3*::*ChR2(L132C;H134R;T159C)*::*YFP(80ng/μl);pmyo-2*::*mCherry]*, **ZX2022**: N2;*zxEx1031[pmyo-3*::*ACR1*::*eYFP(30ng/μl);pmyo-2*::*mCherry]*, **ZX2023**: N2;*zxEx1032[pmyo-3*::*ACR2*::*eYFP(30ng/μl);pmyo-2*::*mCherry]*, **ZX2024**: N2;*zxEx1033[punc-17*::*ACR1*::*eYFP(30ng/μl);pmyo-3*::*mCherry]*, **ZX2026**: N2;*zxEx1034[punc-17*::*ACR2*::*eYFP(30ng/μl);pmyo-3*::*mCherry]*, **ZX2206**: *N2;zxEx1073[pmyo-3*::*ACR1(C102A)*::*eYFP(30ng/μl);pmyo-2*::*mCherry]*, **ZX2207**: *N2;zxEx1074[pmyo-3*::*ZipACR*::*eYFP(30ng/μl);pmyo-2*::*mCherry]*.

All experiments were done using young adult hermaphrodites (picking L4 animals the evening before the experiment).

Animals expressing tools with enhanced light sensitivity (particularly Quint) were cultivated under zero light conditions to avoid stimulation. This included wrapping of NGM plates with aluminum foil and keeping them in a closed incubator. Handling of animals was done under low-level red light using appropriate filter glass.

### Molecular biology

Plasmids pAG54 (pmyo-3::ChR2(H134R)::YFP) [7], pCoS6 (pglr-1::loxP::LacZ::STOP::loxP::ChR2(H134R)::mCherry::SL2::GFP) [48], pCS86 (pmyo-3::ChR2(C128S)::YFP) [8], pCS106 (pglr-1::ChR2(C128S)::YFP) [8], pCS126 (punc-47::ChR2(C128S)::YFP) [8], and pNP259 (pgpa-14::Cre) [48] were described earlier. The plasmid pCS116 (pmyo-3::ChR2(C128S;H134R)::YFP) was generated from pCS86 (pmyo-3::ChR2(C128S)::YFP) by site-directed mutagenesis using the primers oCS222 (5’-GGTCATTCTCATTCGCCTGTCAAACCTGAC-3’) and oCS223 (5’-GTCAGGTTTGACAGGCGAATGAGAATGACC-3’).

A PCR fragment amplified from pCS116 using primers oCS305 (5’-CACCTCACCGGTCATTTGCATTCGCC-3’) and oCS306 (5’-CCGGTGGCCATGGCGGAAGTGGCGCCCCACACAATGCAGCCAATAGCAG-3’) was cloned into pCS126 using the restriction enzymes *Age*I and *Msc*I, resulting in pCS172 (punc-47::ChR2(C128S;L132C;H134R;D156A;T159C::YFP). A fragment of ChR2 including the five point mutations was subcloned into pCS116 with *Age*I and *Pvu*II to generate pCS173 (pmyo-3::ChR2(C128S;L132C;H134R;D156A;T159C)::YFP).

To construct pKE34 (pmyo-3::ChR2(T159C)::YFP) a PCR product was amplified from pChR2(T159C), using primers oKE79 (5`-GTCGTCAATGGCTCTGTACTTGTG-3`) and oKE80 (5`-AGAGCCAAGCCATGCCAGTC-3`) and inserted into pmyo-3::ChR2::YFP with *Bsu*36I and *Dra*III restriction sites.

To generate pKE35 (pmyo-3::ChR2(H134R;T159C)::YFP) two PCRs were performed with oKE79+81 (5`-GCCAATATCAGACACAAGCAGACC-3`) and oKE80+82 (5`-GGGTCTGCTTGTGTCTGATATTGG-3`) from pmyo-3::ChR2(H134R)::YFP and pKE34, respectively, leading to fragments carrying H134R and T159C separately, that were fused and inserted in the pmyo-3::ChR2(H134R)::YFP vector.

To construct pEF 11 (pmyo-3::ChR2(L132C)::YFP) pChR2(L132C) (a gift from E. Bamberg) and pmyo-3::ChR2(H134R)::YFP were restricted with *Dra*III and *Xho*I and the ChR2(L132C) fragment was inserted into the pmyo-3::ChR2(H134R)::YFP backbone. pEF 14 (pmyo-3::ChR2(L132C_H134R_T159C)::YFP) was contructed by fusion PCR amplification generating fragments from pEF 11 with primer pairs oKE79 (5`-GTCGTCAATGGCTCTGTACTTGTG-3`), oAD22rev (5`-GACAGGCGAATGCAAATGACC-3`), and oAD22fw (5`-GGTCATTTGCATTCGCCTGTC-3`), oKE81 (5`-GCCAATATCAGACACAAGCAGACC-3`) and then fused with primer pair oKE79/81 and inserted into pEF11 with *Bsu*36I and *Dra*III restriction sites. pEF15 (pmyo-3::ChR2(C128S;L132C;H134R)::YFP) was generated by fusion PCR with fragments from pEF11 amplified with primer pairs oKE79 (5`-GTCGTCAATGGCTCTGTACTTGTG-3`), oAD23rev (5`-GACAGGCGAATGCAAATGACCGGTGAGGTG-3`), and oAD23fw (5`-CACCTCACCGGTCATTTGCATTCGCCTGTC-3`), oKE81 (5`-GCCAATATCAGACACAAGCAGACC-3`), fused with primer pair oKE79/81 and followed by restriction with *Bsu*36I and *Dra*III and insertion into pEF11.

The plasmid pAB05 (pmyo-3::ChR2(H134R;D156C)::YFP), was generated from plasmid pmyo3::CHOP-2(H134R-g.o.f.)::YFP, by site-directed mutagenesis using primers oST56 (5′-CATGGGTCTGCTTGTGTCTTGTATTGGCACAATTGTGTG-3‘) and oST57 (5′-ACACAATTGTGCCAATACAAGACACAAGCAGACCCATG-3′). The constructs pAB01 (pmyo-3::ACR1::eYFP) and pAB02 (pmyo-3::ACR2::eYFP) were generated via subcloning of ACR1 (pFUGW-hGtACR1-eYFP) and ACR2 (pFUGW-hGtACR2-eYFP) [24] into pmyo-3 vector pDD96.52 (Fire Lab Vector Kit), using restriction enzymes *Eco*RI and *Xba*I. Subloning into the punc-17 vector RM#348p (gift from J. Rand) with restriction enzymes *Hinc*II and *Nhe*I for RM#348p and *Eco*RI and *Xba*I for pFUGW-hGtACR1-EYFP and pFUGW-hGtACR2-EYFP resulted in plasmids pAB03 (punc-17::ACR1::eYFP) and pAB04 (punc-17::ACR2::eYFP).

The plasmid pAB09 (pmyo-3::ACR1(C102A)::eYFP) was generated from plasmid pAB01 (pmyo-3::ACR1::eYFP) by site-directed mutagenesis using primers ACR1_Q5_fwd (5′-GGTGTGCACCGCCCCTATCATGCTGG-3‘) and ACR1_Q5_rev (5′-CAGCTGGCCATTCTGGCC-3′).

The construct pAB10 (pmyo-3::ZipACR::eYFP) was generated via subcloning of ZipACR (pFUGW-ZipACR-eYFP) [38] into the pmyo-3 vector pDD96.52 (Fire Lab Vector Kit), using the restriction enzymes *Bam*HI and *Eco*RI.

For plasmid maps of all optogenetic tools see S3–S15 Data.

### Fluorescence microscopy

Transgenic animals were transferred on 2% agarose pads in M9 buffer (K_2_PO_4_ 20mM; Na_2_HPO_4_ 40mM; NaCl 80mM; MgSO_4_ 1mM) and immobilized with 1μl 50mM NaN_3_ solution (Sigma-Aldrich, Germany) in M9. Expression was analysed using a Zeiss Axiovert 200 or Observer microscope, equipped with a 100W HBO mercury lamp, and a YFP or GFP filterset (AHF Analysentechnik, Germany). Images were obtained with a Axiocam MRm and Axiovision software (Zeiss, Germany), with CoolSNAP HQ2 camera (Photometrics, USA) and MetaVue software (Molecular Devices, USA), or with OrcaFlash 4.0 (Hamamatsu, Germany) and μ-Manager software [49].

### Behavioral experiments

Transgenic animals were cultivated overnight on ATR-supplemented NGM plates: 0.15μl of stock (100mM in ethanol) mixed with 300μl OP-50 bacterial solution and spread on 6cm dishes containing 10ml NGM. Prior to experiments, animals were transferred to unseeded NGM plates, enabling video analysis free from artefacts. Light intensity was adjusted by neutral density filters (AHF Analysetechnik, Germany) and monitored using an optical power meter (PM100, Thorlabs, USA). Recording of body length changes was performed on an Axiovert 40 CFL microscope (Zeiss, Germany) with 10x magnification using a Powershot G9 camera (Canon, USA). For photoactivation, transgenic animals were exposed to light pulses of the respective wavelength (HBO50 light source), controlled via a computer- or Arduino (https://www.arduino.cc)-driven shutter (Sutter Instruments, USA).

For analysis, videos were extracted and individual frames were processed with custom written scripts for ImageJ (National Institutes of Health, USA; https://imagej.nih.gov/ij/index.html [8]) or Matlab (Mathworks, USA) to yield bodylength [13]. Frames yielding false values (e.g. animals coiled) were excluded. For raw data of contraction assays please see S1 Data (depolarizers) and S2 Data (hyperpolarizer). For evaluation of data, bodylength was normalized to recording period prior to illumination. Light pulse protocols, wavelengths, and intensities were specifically adjusted for each experiment as indicated in the figure legends.

For analysis of off-ATR excitability, animals were cultivated in presence of ATR until young adulthood and then transferred to freshly seeded plates without ATR. At specific time points after transfer, body contractions evoked by light stimulation were analyzed.

### Electrophysiology in *C*. *elegans*

For recordings from BWMs animals were immobilized with Histoacryl glue (B. Braun Surgical, Spain) and a lateral incision was made to access neuromuscular junctions along the ventral nerve cord. The basement membrane overlying muscles was enzymatically removed by incubation in 0.5mg/ml collagenase for 10s (C5138, Sigma-Aldrich, Germany). Muscles were patch-clamped in whole-cell mode at 22°C using an EPC10 amplifier with head stage connected to a standard HEKA pipette holder for fire-polished borosilicate pipettes (1B100F-4, WPI, USA) of 4-7-MΩ resistance. For all tools, the bath solution contained: NaCl 150mM; KCl 5mM; CaCl_2_ 5mM; MgCl_2_ 1mM; glucose 10mM; sucrose 5mM; HEPES 15mM, pH7.3 with NaOH, ≈330mOsm. For all depolarizing tools, the pipette solution contained: KCl 120mM; KOH 20mM; MgCl_2_ 4mM; N-tris[Hydroxymethyl]methyl-2-aminoethane-sulfonic acid 5mM; CaCl_2_ 0.25mM; sucrose 36mM; EGTA 5mM; Na_2_ATP 4mM, pH7.2 with KOH, ≈315mOsm. Recordings were conducted at a holding potential of -60mV. For all hyperpolarizing tools, the pipette solution contained Potassium-gluconate 115mM; KCl 25mM; CaCl_2_ 0.1mM; BAPTA 1mM; HEPES 50mM, pH7.3 with 1M KOH, ≈330mOsm. Recordings were conducted at a holding potential of 0mV. Light activation was performed using a LED lamp (KSL-70, Rapp OptoElectronic, Germany; 8mW/mm^2^) and controlled by Patchmaster software (HEKA, Germany). Data were analyzed by Patchmaster software (HEKA, Germany).

### Electrophysiology in HEK293 cells

HEK293 cells were transfected using the ScreenFectA transfection reagent (Waco Chemicals, USA). ATR (Sigma) was added as a stock solution in ethanol at the final concentration of 4μM. Photocurrents from the ACR(C102A) mutant expressed in HEK293 cells (S5i Fig) were recorded 48h after transfection with an Axopatch 200B amplifier (Molecular Devices, USA) using the 2kHz low-pass Bessel filter. The signals were digitized at 5kHz with a Digidata 1440A using pClamp 10 software (both Molecular Devices). Patch pipettes with resistances of 2-5MΩ were fabricated from borosilicate glass. The pipette solution contained (in mM): K-gluconate 135, MgCl_2_ 2, HEPES 20, pH7.2. The bath solution contained (in mM): NaCl 150, CaCl_2_ 1.8, MgCl_2_ 1, glucose 5, HEPES 10, pH7.4. Continuous light pulses were provided by a Polychrome V light source (T.I.L.L. Photonics GMBH, Germany) at the half-bandwidth 15nm in combination with a mechanical shutter (Uniblitz Model LS6, Vincent Associates, USA; half-opening time 0.5ms). The quantum density at the focal plane of the 40x objective lens was 7.7mW/mm^2^. All measurements were carried out at room temperature (25°C).

### Statistics

Data were analyzed in OriginPro 2015G (OriginLab Corporation, USA). Data are given as means±SEM. Significance between data sets after two-tailed Student’s t-test or ANOVA is given as p-value (* p ≤ 0.05; ** p ≤ 0.01; *** p ≤ 0.001), the latter after Bonferroni′s multiple comparison test, or Tukey’s post-hoc test.

## Results

### Characterization of ChR2 variants for long-term depolarization

Several ChR2 variants have been established for long-term depolarization, exhibiting a slowed-down photocycle due to modifications within the DC gate. We attempted to overcome limitations such as desensitized states after channel-closing [8] and dependence on continuous presence of ATR by combining known point mutations. In *C*. *elegans*, ChR2(H134R) had shown improved expression, reduced desensitization, and sustained excitability after removal of ATR [27, 50]. Thus, we included the H134R mutation in all tools generated. In double, triple, and quintuple mutants we combined H134R with C128S, D156A or D156C, all slowing down kinetics to various degrees [35, 51, 52], with L132C for increased Ca^2+^ conductance [32], and with T159C for increased plasma membrane insertion or conductivity [21] (Table 1; values for Mac and Arch were calculated from experiments published in [6**]**).

**Table 1:** Summary of results.

Tool		Change in body length (tool expressed in BWM) [%]	τ_relax_ [s]	Light sensitivity	Repeatabilityof stimulation	off-ATR excitability	Advantages of the most significant opsins screened
**DEPOLARIZERS**							
**ChR2(H134R)**		**-14.9**	**1.1**	**-**		**++**	
**ChR2(C128S)**		**-14.6**	**181.7**	**++**	**-**	**-**	
**ChR2(L132C)**	**CatCh**	**-13.5**	**0.7**	**o**			
**ChR2(T159C)**		**-6.4**	**0.3**				
**ChR2(C128S;H134R)**		**-14.6**	**841.3**	**++**	**+**	**++**	
**ChR2(H134R;D156C)**		**-14.3**	**527.8**	**++++**	**++**		
**ChR2(H134R;T159C)**		**-14.7**	**0.6**				
**ChR2(C128S;L132C;H134R)**		**-10.7**	**1253.9**	**+++**			
**ChR2(L132C;H134R;T159C)**		**-16.3**	**2.3**	**+++**			
**ChR2(C128S;L132C;H134R;D156A;T159C)**	**Quint**	**-12.8**	**45370.1**	**+++**		**+**	**very slow closing kinetics high operational light sensitivity good off-ATR excitability**
**HYPERPOLARIZERS**							
**ACR1**		**+6.1**	**0.57**	**+++++**	**+**		**high photocurrents high operational light sensitivity red-shifted action spectrum**
**ACR2**		**+4.8**	**0.14**	**+++++**	**+**		**high photocurrents high operational light sensitivity high temporal precision**
**ACR1(C102A)**		**+3.4**	**26.04**	**+++++**			
**ZipACR**	**PsuACR_973**	**+4.5**	**0.15**	**+**	**+**		
**NpHR**		**+3.5**	**0.16**	**-**	**+**		
**Mac**		**+3.9**	**0.09**		**+**		
**Arch**		**+4.2**	**0.24**		**+**		

**Fig 1:**
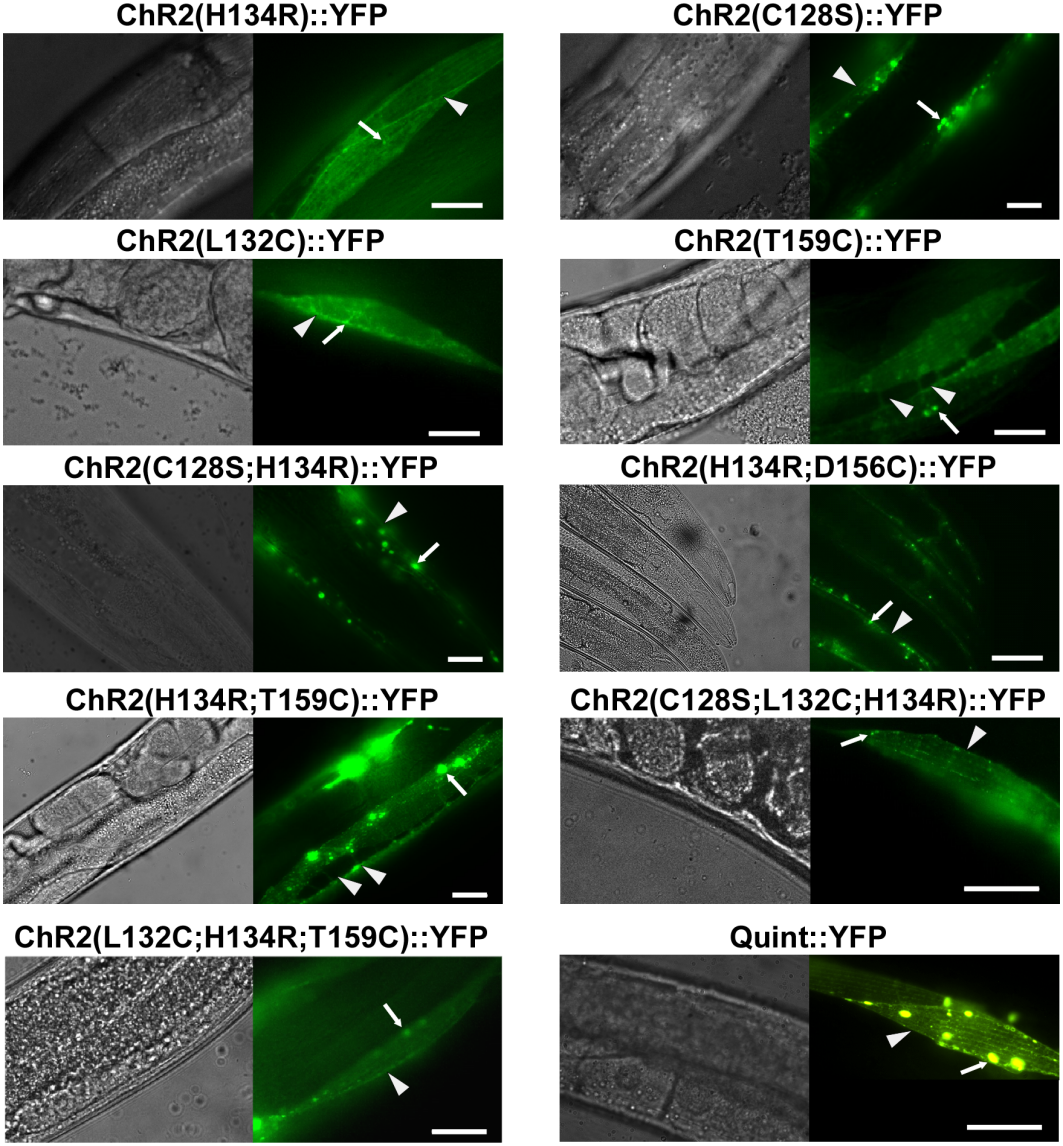
ChR2 variants expressed in body-wall muscle cells localize to membranes. DIC images (left panels) and fluorescence micrographs (right panels) showing expression of ChR2(H134R)::YFP, ChR2(C128S)::YFP, ChR2(L132C)::YFP, ChR2(T159C)::YFP ChR2(C128S;H134R)::YFP, ChR2(H134R;D156C)::YFP, ChR2(H134R;T159C)::YFP, ChR2(C128S;L132C;H134R)::YFP, ChR2(L132C;H134R;T159C)::YFP, and Quint::YFP in body-wall muscle cells of *C*. *elegans*, as indicated. Arrows mark aggregates, arrowheads mark plasma membrane. Scale bar is 10 μm.

YFP-tagged versions of the proteins were expressed in body-wall muscles (BWMs), and after screening numerous transgenic lines, those with low-mosaic, robust and (mostly) plasma membrane localized expression were chosen for experiments (see Methods / *C*. *elegans* culture and transgenic animals), aiming to provide a comparison and quantification of light-evoked effects: ChR2(C128S;H134R), ChR2(H134R;D156C), ChR2(H134R;T159C), ChR2(C128S;L132C;H134R), ChR2(L132C;H134R;T159C), and Quint (ChR2(C128S;L132C;H134R;D156A;T159C)) all localized to the plasma membrane (Fig 1), including muscle arm protrusions (S1 Fig), with some localization in intracellular membranes to variable degrees. Expression levels, as judged by fluorescence, were comparable to those of the single-mutation variants ChR2(H134R), ChR2(C128S), ChR2(L132C), or ChR2(T159C) (Fig 1).

**Fig 2:**
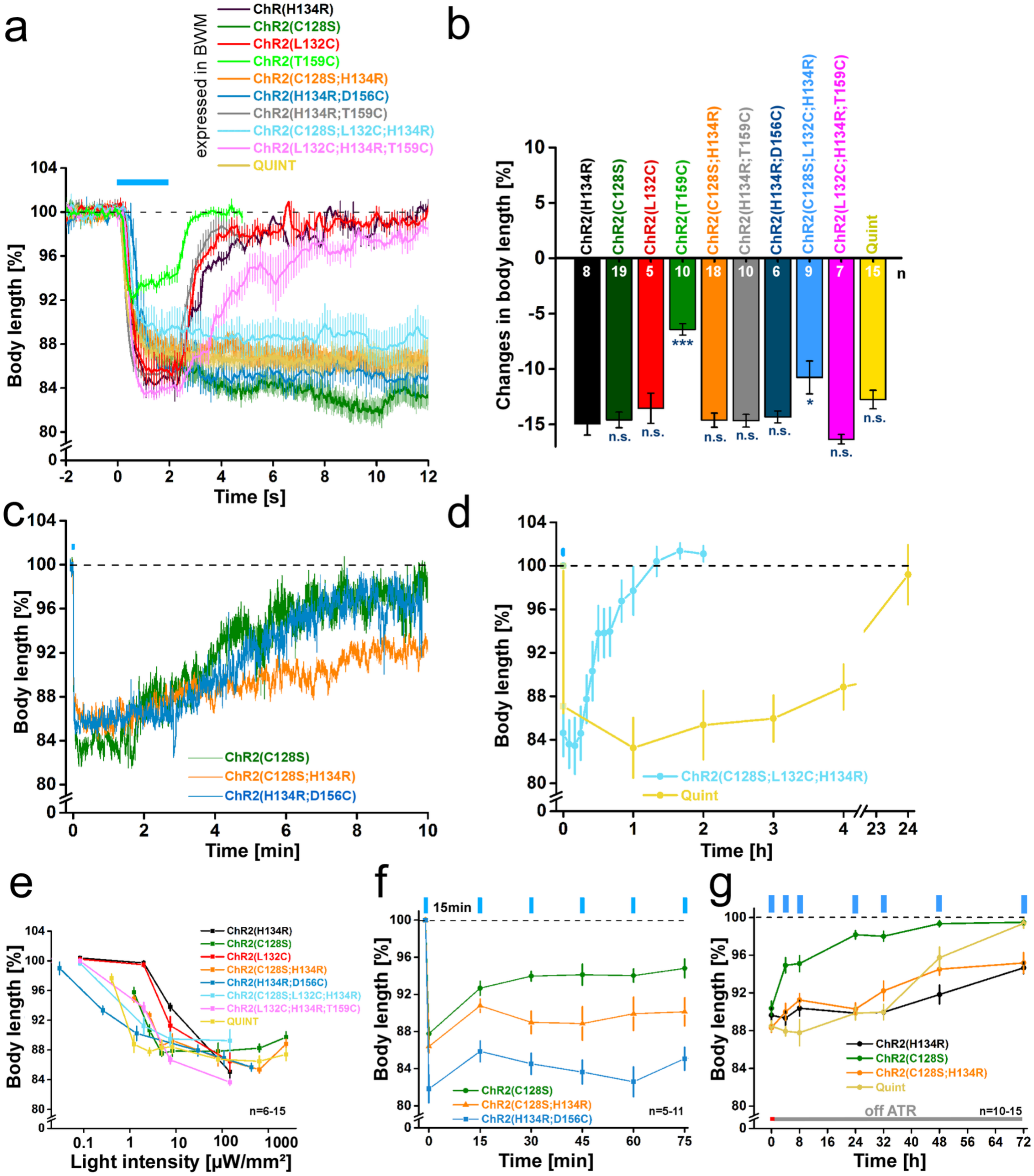
ChR2 variants expressed in body-wall muscle cells enable prolonged depolarization and body contractions. **(a)** Body length of animals expressing ChR2(H134R)::YFP, ChR2(C128S)::YFP, ChR2(L132C)::YFP, ChR2(T159C)::YFP ChR2(C128S;H134R)::YFP, ChR2(H134R;D156C)::YFP, ChR2(H134R;T159C)::YFP, ChR2(C128S;L132C;H134R)::YFP, ChR2(L132C;H134R;T159C):.YFP, and Quint::YFP in body-wall muscle cells of *C*. *elegans* during and after a blue light stimulus (0.2 mW/mm^2^; 1 or 2 s; 450–490 nm; n = 5–19). **b)** Maximal changes in body length of all depolarizers tested. Shown is the mean normalized change in body length (± SEM) relative to the initial length of the animal. Significance given refers to ChR2(H134R): *p<0.05, ***p<0.001. **(c)** Same as in a) but displayed over 600 s for ChR2(C128S)::YFP, ChR2(C128S;H134R)::YFP, and ChR2(H134R;D156C)::YFP (n = 7–19). **(d)** Body length of animals expressing ChR2(C128S;L132C;H134R) (n = 5–6; 2 s illumination; 470 nm; 1 mW/mm^2^) or Quint (n = 8–14; 5 s illumination; 450–490 nm; 2.4 mW/mm^2^) up to 24h after a blue light stimulus (450–490 nm). **(e)** Dependence of body contractions on light intensity in strains listed in (a). Reductions in body length were recorded in response to 1, 2 or 5 s light stimuli (450–490 nm) of light intensities in the range of 0.03–2.41 mW/mm^2^ (n = 6–15). **(f)** Repeated stimulation of animals expressing ChR2(C128S)::YFP, ChR2(C128S;H134R)::YFP, and ChR2(H134R;D156C)::YFP. 2 s blue light pulses (0.2 mW/mm^2^ ((C128S), (C128S;H134R)) or 1 mW/mm^2^ (H134R;D156C); 450–490 nm; n = 5–11) were presented with ISI of 15 min. **(g)** Off-ATR excitability of animals up to 72 h after placing them on fresh NGM plates in absence of ATR (5s; 2.4 mW/mm^2^; 450–490 nm; n = 10–15). Shown is the mean normalized body length (± SEM) calculated relative to the initial length of the animal; n = number of animals. Blue bar indicates illumination period.

Photo-triggered body contractions (S2 Fig) were compared to ChR2(C128S) as the previous benchmark for long-term depolarization in *C*. *elegans* [8]. All strains were illuminated with blue light (1 or 2s; 450-490nm) and contractions were recorded as an indirect measure for the extent of muscle depolarization, while their duration reported on closing kinetics. Illumination of the double mutants ChR2(H134R;T159C), ChR2(H134R;D156C), and ChR2(C128S;H134R) resulted in strong body contractions of -14.7±0.6%, -14.3±0.5%, and -14.6±0.6%, respectively (Fig 2a and 2b). The triple mutant ChR2(C128S;L132C;H134R) led to the smallest contractions of all variants with multiple mutations (-10.8±1.5%; S1 Video), while ChR2(L132C;H134R;T159C) led to the strongest contractions (-16.3±0.4%), featuring a distinct increase compared to the single mutant ChR2(L132C) (-13.5±1.4%). Finally, Quint evoked notable contractions of -12.8±0.8%. Generally, peak contractions were comparable to those evoked by ChR2(H134R) (-14.9±1.0%) and ChR2(C128S) (-14.6±0.7%).

After light was turned off animals expressing the triple mutant ChR2(L132C;H134R;T159C) relaxed their body with a slight delay (τ_relax_ value of 2.31±0.10s, compared to 1.08±0.06s in ChR2(H134R); Fig 2a; Table 1). In all other mutant combinations, with the exception of ChR2(H134R;T159C) (τ_relax_: 0.57±0.03s), the delay in channel closing was much more pronounced and they showed long-lasting contractions. For ChR2(C128S;H134R), ChR2(H134R;D156C) and ChR2(C128S;L132C;H134R), time-dependent recovery was detectable in the minute-range (Fig 2c and 2d). Compared to ChR2(C128S) (τ_relax_: 182±1.5s) contractions lasted considerably longer for ChR2(H134R;D156C) (528±12s), ChR2(C128S;H134R) (841±35s), and ChR2(C128S;L132C;H134R) (1254±292s), indicating further decelerated closing kinetics. Thus, effects that delay channel closing in H134R and C128S single mutants may partly add up, or even influence each other in a synergistic manner. Extremely delayed relaxation was obtained for Quint (τ_relax_: ≈45,000 ± 25,000 s). Here, body contractions began to cease only after ≈4 hours (Fig 2d) and it took 24 hours until return to baseline. In fact, Quint may not be closing at all, and the recovery from contraction could simply be caused by turnover of the protein. Closing of Quint with yellow or red light, as is possible for C128S [8], was not achievable (data not shown). Due to high light sensitivity and slow recovery complementary electrophysiological recordings could not be performed since the ambient light required for dissection of the animals, even when we worked under minimal red-light conditions, already pre-activated these rhodopsins (for an action spectrum of Quint and in particular in response to red light of intensities used also for dissection of animals for electrophysiology, see S4 Fig). Animals showed increasing contraction (12%) already after 15 s of illumination, i.e. after a much briefer period than would be required for dissection.

The delayed closing kinetics should indirectly also increase the operational light sensitivity of these ChR2 variants, respectively the minimal light intensity required to evoke full contractions *in vivo*: As the proteins accumulate in the open state, reduced light intensities suffice to photoactivate a larger fraction of ChR2 molecules, the slower the photocycle (transition from P520 state to P480 state [53, 54]). To analyze this, we illuminated at different light intensities (0.03μW/mm^2^–2.41mW/mm^2^). While ChR2(H134R) requires intensities of 147μW/mm^2^ to elicit maximal contractions (Fig 2e; S3a Fig), ChR2(C128S;H134R) still evoked full effects at just 4.9μW/mm^2^, similar to ChR2(C128S) (S3b and S3d Fig). ChR2(H134R;D156C) turned out to be particularily useful for low-light applications: 0.26μW/mm^2^ sufficed to evoke relevant contractions (Fig 2e; S3e Fig) and it was ≈3x more light sensitive than ChR2(C128S;H134R). For Quint, the minimum light intensity to evoke relevant contractions was 1.22μW/mm^2^, making it ≈120x more light-sensitive than ChR2(H134R) (Fig 2e; S3h Fig).

In HEK293 cells, ChR2(L132C) had shown an increased light sensitivity [32], presumably due to enhanced activation of voltage-gated Na^+^ channels. However, in *C*. *elegans* L132C only led to a minor increase in light sensitivity compared to ChR2(H134R) (Fig 2e). This may be due to the lack of Na^+^ channels in *C*. *elegans*. Both (H134R) and (L132C) required light intensities of 7.5μW/mm^2^ before showing substantial contractions. Maximal effects were reached at 147μW/mm^2^ with body length decreases by -14.9±1.0% (H134R) and -13.5±1.4% (L132C) (S3a and S3c Fig). In contrast, the triple mutants ChR2(C128S;L132C;H134R) and ChR2(L132C;H134R;T159C) achieved substantial contractions already at 2μW/mm^2^, also reaching a maximum at 147μW/mm^2^ (Fig 2e; S3f and S3g Fig).

ChR2(C128S) and other ‘slow’ ChR2 variants enter long-lived desensitized states after channel closing, which limits their utility for repetitive photostimulation. This allows ongoing depolarization only at reduced light intensity, thus reducing efficiency [55]. As desensitized states were reduced for ChR2(H134R), we wondered if the desensitization would be less prominent for the (C128S;H134R) and (H134R;D156C) double mutants. Therefore, we presented repeated light stimuli (2s; 450-490nm; 200μW/mm^2^) at interstimulus intervals (ISIs) of 15 minutes (Fig 2f). Starting with contractions of -12.2±0.7%, ChR2(C128S) reached a 50% reduced plateau activity with the third stimulus (-6.0±0.5%). In contrast, photostimulation of ChR2(C128S;H134R) caused an initial contraction of -13.6±0.7%, that was reduced by only 19% (-11.0±1.2%) at the third stimulus. A similar decrease of 15% was observed with ChR2(H134R;D156C) (-18.2±1.5% initial contraction, reduced to -15.5±1.1% at third stimulus). These results demonstrate that H134R indeed confers a reduced tendency for desensitization, also when combined with other mutations. Thus, ChR2 variants for long-term depolarization are potentiated by the H134R mutation.

Finally, we analyzed the off-ATR excitability. Use of ChR2(C128S) for long-term depolarization requires ongoing supplementation of ATR [8]. This property limits applicability specifically in organisms that do not provide sufficient endogenous amounts of ATR, or which do not allow sufficient uptake of exogenous ATR (e.g. in eggs, or in certain developmental stages like the non-feeding dauer larva of *C*. *elegans*, and in experiments where depolarization needs to be achieved for hours or days). ATR removal reduced contractions of ChR2(C128S)-expressing animals within four hours (and even earlier with more frequent repeated stimulation [8]), while in ChR2(H134R)-expressing animals effects only started to decay slightly after 32 hours (Fig 2g). Similarly, full contraction effects were found until 24 and 32 hours post ATR removal for ChR2(C128S;H134R) (83% of initial effect) and Quint (87% of initial effect). Quint stopped responding within 72 hours while ChR2(C128S;H134R) still evoked significant contractions (-4.8±1.1%) at this timepoint, as did ChR2(H134R) (-5.3±0.7%). Hence, it appears likely that the augmenting effects of the H134R mutation are in part due to stabilization of the ATR-Schiff base. Consequently, such double mutants are likely preferable tools for optogenetic long-term depolarization when ATR supplementation is limited.

### Photoinhibition of excitable cells by ACRs leads to strong behavioural effects and photocurrents

**Fig 3:**
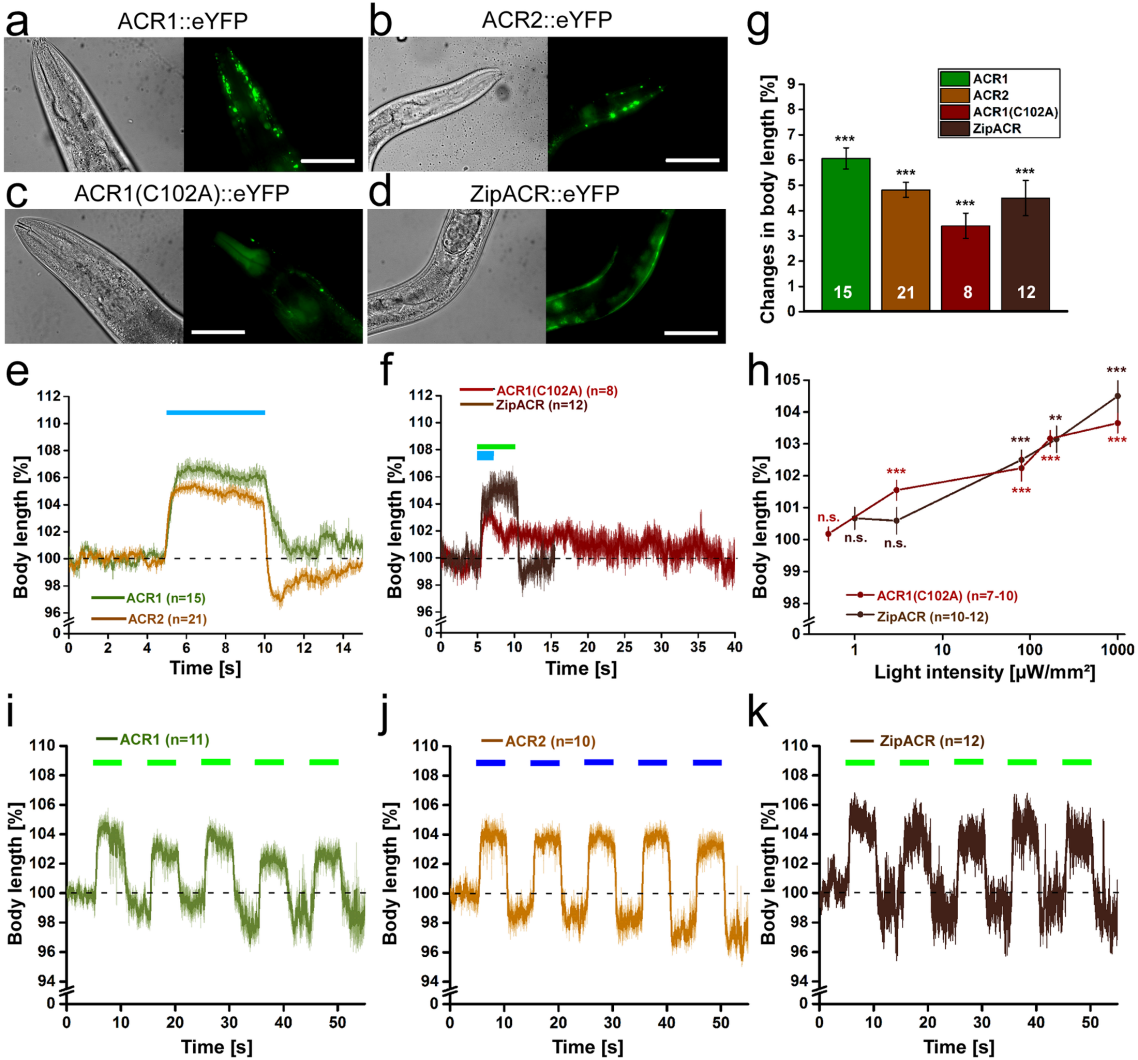
ACRs mediate strong hyperpolarization and body elongation. Expression of ACR1::eYFP **(a)**, ACR2::eYFP **(b)**, as well as ACR1(C102A)::eYFP **(c)** and ZipACR::eYFP **(d)** in body-wall muscle cells of *C*. *elegans*. Scale bar is 50 μm. **(e)** Body length of animals expressing ACR1 or ACR2 during and after a 5 s light stimulus (1 mW/mm^2^; 470 nm). Shown is the mean normalized body length (± SEM) relative to the initial length of the animal. **(f)** Body length during and after a light stimulus (1 mW/mm^2^) of animals expressing ACR1(C102A) (470 nm, 2 s) or ZipACR (520 nm, 5 s). **(g)** Maximal changes in body length induced by the tested hyperpolarizers. Shown is the mean normalized change in body length (± SEM) relative to the initial length of the animal. **(h)** Light intensity dependence of the body elongation of animals expressing ACR1(C102A) or ZipACR in body-wall muscle cells. **(i)** Body length in response to repetitive photostimulation (5 s, 5 s ISI, 80 μW/mm^2^, 470 or 520 nm) of ACR1, ACR2 **(j)** or ZipACR **(k)** in animals expressing the respective channel in body-wall muscles; n = number of animals. Blue and green bars indicate illumination period. *p<0.05, **p<0.01, ***p<0.001.

Next, we turned to new rhodopsin tools for neuronal inhibition, namely the natural anion channel rhodopsins ACR1, ACR2, and ZipACR, as well as the ACR1(C102A) step-function variant. All variants were expressed as eYFP fusion proteins in BWMs. Fluorescence was visible in the plasma membrane of BWMs (Fig 3a–3d). Illumination of ACR1 and ACR2 (5s; 470nm; 1mW/mm^2^) resulted in the strongest body relaxation that we have ever observed, with maxima of 6.1±0.4% for ACR1 (Fig 3e and 3g), while ACR2 let to maximal elongations of 4.8±0.3% (Fig 3e and 3g). For comparison, Arch mediated maximal effects of 4.2±0.3% (at 568 nm) at saturating light levels [6]. Elongations ended right after termination of illumination and body length returned to baseline levels within 1-2s (τ_’relax’_: 0.57±0.03s (ACR1), 0.14±0.02s (ACR2)). Values are similar to τ_’relax’_ values that we previously observed for NpHR (τ_’relax’_: 0.16±0.05s), Mac (τ_’relax’_: 0.09±0.03s), and Arch (τ_’relax’_: 0.24±0.04s; all expressed in BWMs) [6].

Illumination of ACR1(C102A) (2s, 470nm, 1mW/mm^2^) and ZipACR (5s, 520nm, 1mW/mm^2^) expressed in BWMs led to body elongations of 3.4±0.5% and 4.5±0.7%, respectively (Fig 3f and 3g; S2 Video). In contrast to the large currents reported for expression in HEK293 cells [38], ZipACR did not induce stronger behavioral effects than other ACRs in *C*. *elegans*. Its faster kinetics [38] did not obviously shorten the time BWMs require to contract after light-off (τ_’relax’_: 0.15±0.03s) compared to ACR2. ACR1(C102A) led to the expected dramatic decrease in channel closing (τ_’relax’_: 26.04±4.62s), however, the maximal elongation was reduced by 56% compared to ACR1. Interestingly, animals expressing ACR2 or ZipACR often exhibited a slight body contraction right after cessation of the photostimulation (Fig 3e and 3f). This might represent the action of Cl^-^-pumps extruding negative charge from cells or indicate a depolarizing effect as a rebound reaction. The slight contraction might also be provoked by the fast speed of the response in ACR2 and ZipACR while it seems to be independent of its strength, since the effect was not observed with ACR1 which evoked strong body elongations just like ACR2.

Experiments at low light intensities revealed a high operational light sensitivity for ACR1 and ACR2, showing about half-maximal elongations at 75μW/mm^2^ when ACR1 (43% of maximum at 1mW/mm^2^) and ACR2 (56% of maximum at 1mW/mm^2^) were expressed in cholinergic neurons (S5d, S5e and S5f Fig). In agreement with the action spectrum of ACR1 being slightly red-shifted [24], ACR1- (but not ACR2-) expressing animals could also be stimulated with green light (520nm) at low light intensities (3.6μW/mm^2^; S5d Fig). At 80μW/mm^2^ about half-maximal elongations were also reached for ZipACR (55%) and ACR1(C102A) (62%) (Fig 3h; S5g and S5h Fig). However, in contrast to ACR1 and ACR2, ZipACR caused no obvious relaxation effects at lower light intensities. One interesting feature of step-function opsins is the ability of channel closure by red-shifted light. Indeed, ACR1(C102A) expressed in HEK293 cells showed a partial and rather slow reduction in currents after red light exposure (620-660nm; maximum: 640nm) following activation at 515nm (S5i Fig).

Repeated photostimulation of ACR1 (520nm; Fig 3i; S5j Fig), ACR2 (470nm; Fig 3j; S5k Fig), or ZipACR (520nm; Fig 3k) (5s; 80μW/mm^2^, ISI 5s) demonstrated stability during recurrent activation and no obvious desensitization. During dark intervals, we noted a trend to lower baseline body length. Possibly this was due to alterations in cellular Cl^-^-concentration that could not immediately be balanced by intrinsic Cl^-^-pumps. For expression and functional analysis of ACR1 and ACR2 in cholinergic neurons, see S5 Fig and S3 Video.

A more recently generated, improved ChloC (iChloC [36]) was not tested, and neither were iC1C2 and iC++, as we could estimate from the literature that the currents they generate fall behind those of the ACRs [24, 37]; this does not imply that these proteins may not work well in *C*. *elegans*.

**Fig 4:**
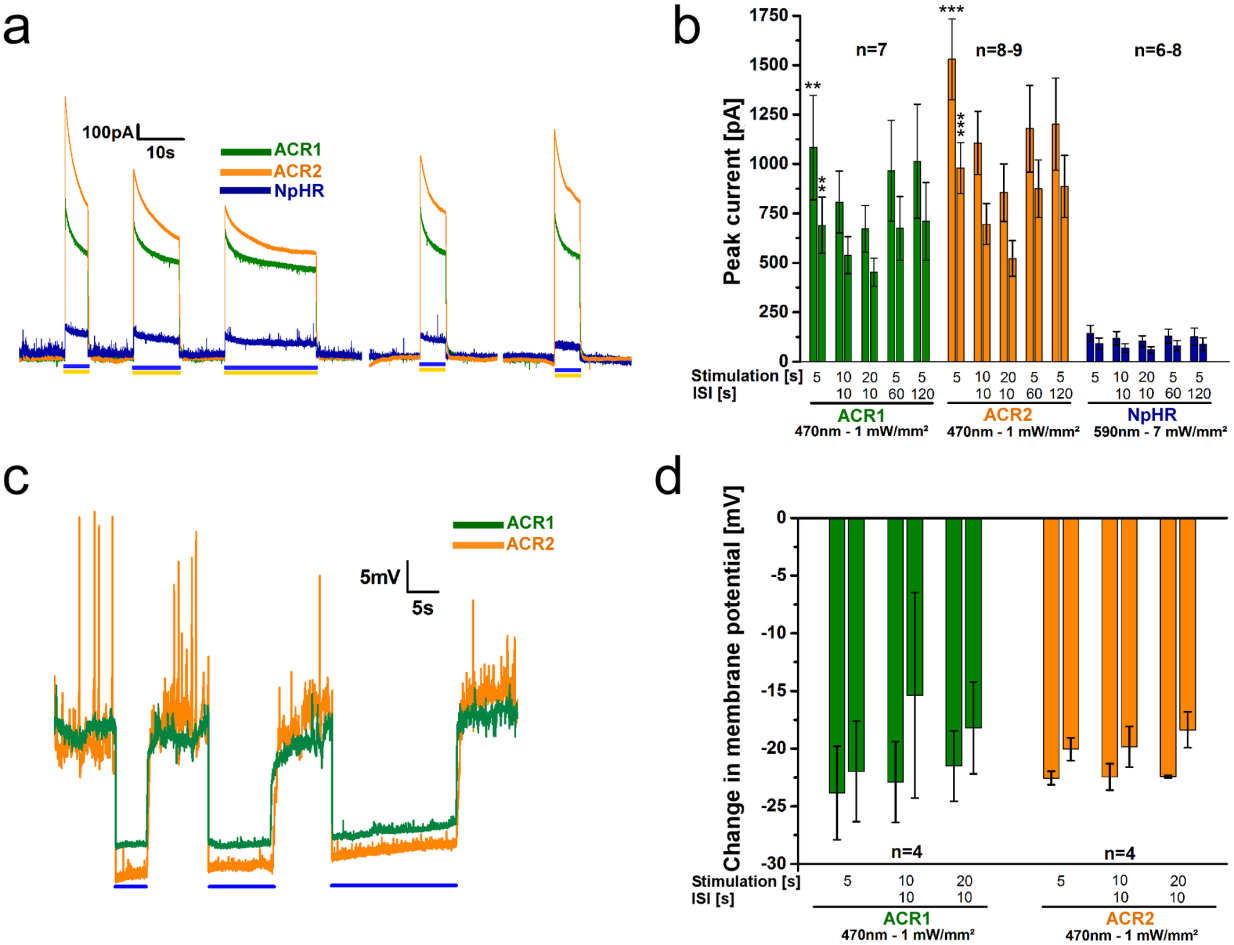
ACR1 and ACR2 mediate large photocurrents and hyperpolarization in patch-clamped BWMs. **(a)** Example current traces of body-wall muscle cells expressing ACR1, ACR2, or NpHR during 5 s, 10 s, and 20 s photostimulation (blue or yellow light, as indicated by bars) with 10 s ISIs. **(b)** Group data of experiments described in a). The paired bars at each time point represent peak currents (1^st^ bar) and plateau currents (2^nd^ bar). Significance refers to NpHR. **(c)** As in a), but showing voltage traces, before, during and after 5 s, 10 s, and 20 s photostimulation with 10 s ISI. **(d)** Group data for experiments shown in c). The paired bars at each time point represent peak (1^st^ bar) and plateau (2^nd^ bar) changes in the membrane potential. ACRs were illuminated at 470 nm (1 mW/mm^2^), NpHR at 590 nm (7 mW/mm^2^); n = number of animals. Displayed are mean ± SEM of currents or voltage in b) and c). **p<0.01, ***p<0.001.

To investigate function of the most promising hyperpolarizers more precisely, we measured photocurrents of ACR1 and ACR2 and for comparison NpHR by patch-clamp recordings from BWMs. Photostimulation with blue light (470nm, 5s, 1mW/mm^2^) evoked large outward peak currents of 1083±264pA (τ_off_: 0.23±0.05s) and 1530±204pA (τ_off_: 0.10±0.02s) for ACR1 and ACR2, respectively (Fig 4a and 4b). These currents were about 10-fold larger than those obtained for NpHR at its excitation maximum of 590nm (143±41pA; 5.3mW/mm^2^). Ratios of currents at the end of the photostimulation and the initial peak currents were almost identical for all three hyperpolarizers (≈64%). In case of the pump NpHR, the decrease in currents is most likely caused by a progressive increase of inactivated proteins. In case of the channels (ACRs) it may be a combination of desensitization and, due to the strong conductivity, also a drop in the electrochemical potential driving Cl^-^-influx. To test the influence of stimulus length and repeated stimulation, we conducted a protocol starting with light stimuli of 5, 10, and 20s separated by ISIs of 10s, followed by longer ISIs of 60 and 120s with 5s of stimulation each. All three rhodopsins could be repetitively stimulated. The last peak current in the sequence reached 94, 79, and 88% of the initial peak for ACR1, ACR2, and NpHR, respectively (Fig 4b). For ACRs, the currents continued to decay even after 20s of photostimulation, but depending on the duration of the ISI, the next peak current recovered towards initial values. This is in line with dissipation of the Cl^-^-gradient during the light stimulus, and implies that it is counteracted by Cl^-^-pumps (with net efflux during dark periods). As this should affect membrane voltage, we also recorded the membrane potential (Fig 4c and 4d). Spontaneous action potential bursts [56, 57] were suppressed by ACR activation. Also here we observed a hint of the action of Cl^-^-efflux pumps, particularly during dark intervals, as the baseline potential gradually increased (see S6 Fig for averaged, filtered traces, showing baseline depolarization more obviously), as expected for extrusion of negative charge.

Our behavioral and electrophysiological results support previous reports for HEK293 cells [40], suggesting stable effects and high photocurrents mediated by ACRs, making them very effective for optogenetic (shunting) hyperpolarization, at least at depolarized potentials. For action near the Cl^-^ equilibrium potential, NpHR (as a pump) can provide further activity, as it operates mostly independent of the membrane potential.

## Discussion

**Fig 5:**
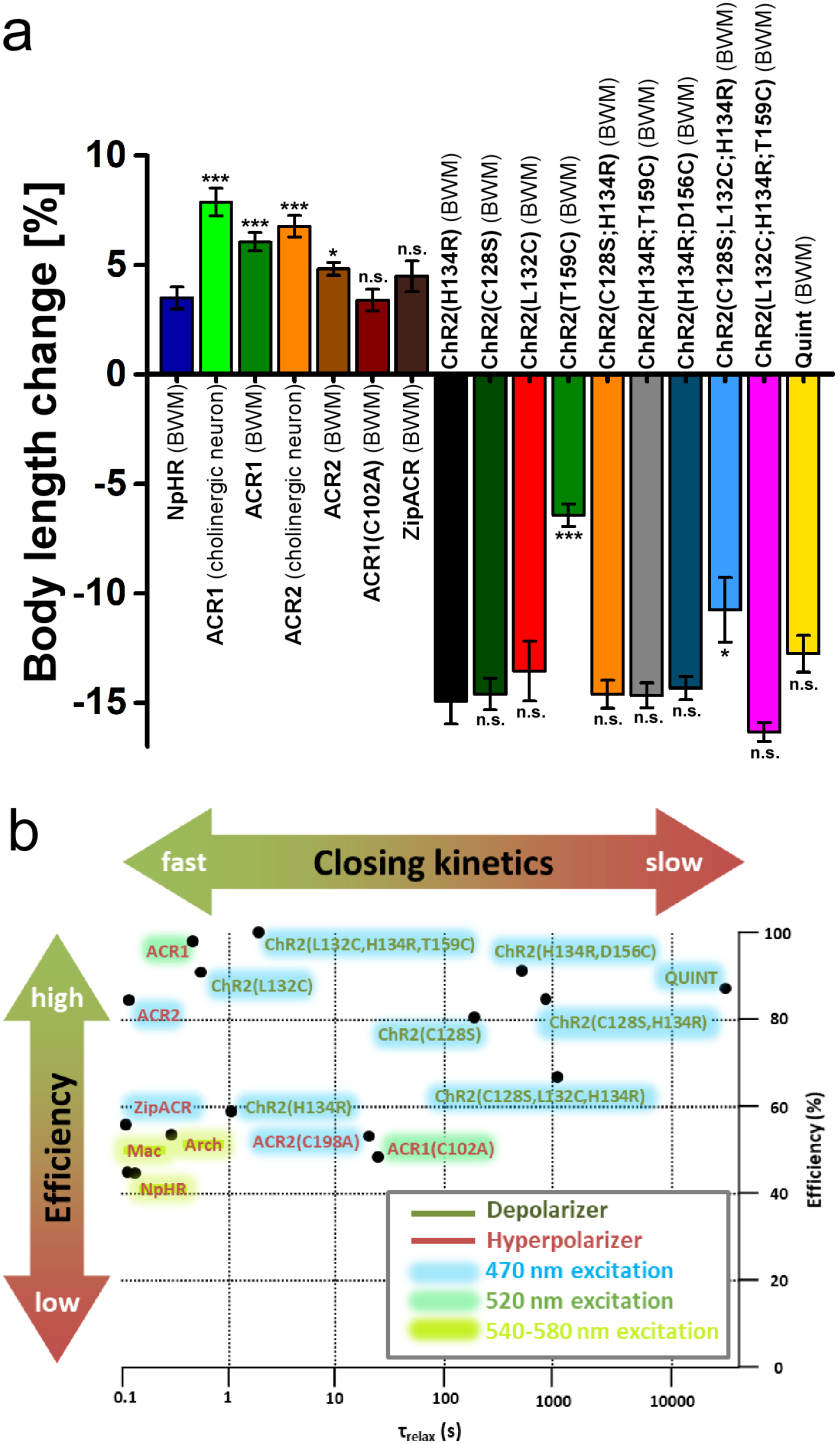
Evaluation of de- and hyperpolarizers characterized in this paper. **a)** Changes in body length induced by de- and hyperpolarizers. Shown is the mean normalized change in body length (± SEM) relative to the initial length of the animal; n = number of animals. Statistical differences for hyperpolarizers are referring to NpHR, statistical differences for depolarizers are referring to ChR2(H134R). **b)** Scheme of optogenetic de- and hyperpolarizers expressed in BWMs of *C*. *elegans* classified by closing kinetics (τ_relax_) and efficiency. The efficiency was determined as follows: Depolarizers—Relative comparison of contractions, induced by the respective tool, at 200 μW/mm^2^ to the maximum possible contraction; Hyperpolarizers—Relative comparison of relaxations, induced by the respective tool, at 1 mW/mm^2^ to the maximum possible relaxation. Hence, efficiency not only refers to the maximum possible changes in body length upon light saturation, but also includes information about the tool’s light sensitivity. Therefore, some tools receive lower efficiencies, though they exhibit comparable maximum effects. Color shades indicate the light color of the respective tool’s excitation wavelength.

Due to its reliability and relatively fast kinetics, H134R –the ‘standard’ ChR2 –is widely used as an optogenetic tool for depolarization of excitable cells. However, the demanding light requirements and progressive inactivation prevent its use for long-term experiments. ChR2(C128S) was established as a variant with slowed closing kinetics that allows prolonged depolarization over several minutes following a single short photostimulus. Yet, the requirement of continuous supplementation of the cofactor ATR and reduced effects during repetitive stimulation, as a consequence of the accumulation of desensitized states, limited its applicability. To bypass these limitations, we combined previously known mutations and characterized the resulting ChR2 variants for favorable properties in long-term depolarization (Fig 5a; Table 1).

Compared to ChR2(C128S), contractions evoked by ChR2(C128S;H134R) lasted slightly longer, while exhibiting similar operational light sensitivity. Yet, ChR2(C128S;H134R) exhibited superior off-ATR excitability, allowing to elicit strong contraction in BWMs for at least 24h after ATR removal, while effects for ChR2(C128S) ceased within 4h. Repetitive stimulation of ChR2(C128S;H134R) elicited higher contractions, indicating that it is less likely to be lost to desensitized states. Even higher effects during repetitive stimulation were found for ChR2(H134R;D156C). Given that H134R remains fully activatable after 32h off ATR, we expect that H134R;D156C will show similar stability.

The different properties of ChR2(C128S) and ChR2(C128S;H134R) likely result from the H134R mutation. The open state life time in ChR2(H134R) is increased compared to ChR2(wt) (17.9 vs. 13.5ms; [58]). Slowing effects on channel closing by C128S and H134R may thus add up (in a non-linear manner) in the double mutant. In a previous study it was proposed that mutants of C128 are more likely to lose their ATR from the chromophore binding pocket after channel closing, i.e. in specific states reached from the P480 state [30]. Reloading the apoprotein with ATR may be time-consuming or may be hampered after an initial photoactivation, which could explain the longevity of the ‘lost’ states. However, the need for reloading in C128S would also explain the need for ongoing presence of ATR. Why H134R affects the stability of the ChR2-ATR Schiff base can only be speculated. Nonetheless, charge/polarization effects may be involved. Yet, this hypothesis requires more direct biophysical analyses of this ChR2 variant. With its improved off-ATR excitability and response to repetitive stimulations, ChR2(C128S;H134R) might be favored over C128S, at least when ATR supplementation is a limiting factor, like in *D*. *melanogaster* or *C*. *elegans*, and when strong depolarizations are to be induced for more than a single photocycle. ChR2(H134R;D156C) may be even more useful in this context, given the stronger depolarization induced during the first 75 minutes.

The quintuple mutant Quint exhibited the slowest closing kinetics of all ChR2 variants known so far (including ChR2-XXL), causing depolarization of BWMs for many hours following a single 1s light stimulus and only returning to baseline values after about 24 hours. Unlike for the step-function opsins, we could not close the channel by using yellow light (data not shown), and it cannot be excluded that the behavioral effects seized only when the protein was degraded. Nonetheless, Quint extends temporal limits for minimal light-invasive, optogenetic depolarization. In line with drastically slowed closing kinetics, the operational light sensitivity was increased, evoking full contractions in BWMs at light intensities as low as 1.22μW/mm^2^ (ca. 1/10^th^ light intensity of the sun on an overcast day, while light intensities used for ChR2(H134R) experiments correspond to a sunny summer day [59]), i.e. ≈4-fold lower than for ChR2(C128S) and ≈120-fold lower than for ChR2(H134R). When stimulation of excitable cells for hours or days is intended and at low expression levels, Quint would be a good choice, possibly also in other model systems.

The ACRs are an important extension to the repertoire of inhibitory optogenetic tools in *C*. *elegans*, because they combine channel properties and fast action. They feature extraordinarily high photocurrents in BWMs at low light intensities, even if this necessitates keeping in mind the corresponding alterations in cellular Cl^-^ concentrations. Their anion selectivity and high temporal precision make ACRs very efficient tools to inhibit neuronal activity with light. Furthermore, the red-shifted action spectrum of ACR1 facilitates a selective combination with blue-light activated optical tools. ACR1(C102A) demonstrated sustained and long-lasting effects in *C*. *elegans*, resembling those of the common depolarizing step-function mutants. This is a useful feature for long-term hyperpolarization applications.

In sum, we generated and characterized several new ChR2 variants and ACRs in *C*. *elegans* for either depolarization or hyperpolarization, complementing the optogenetic toolbox (for an overview, see Fig 5b). Particularily ChR2(C128S;H134R), ChR2(H134R;D156C), and Quint bypass limitations of optogenetic long-term and repetitive depolarization, particularly in animals lacking endogenous ATR. Furthermore, we could establish ACRs as powerful tools for fast optogenetic inhibition in the nematode. Together, these optogenetic tools allow a straightforward, easy and efficient manipulation of neuronal activity, likely also in other systems (however, we emphasize that this will have to be re-tested in the system of interest, as opsin expression is not universally similar in all hosts). They are particularly useful when only low light intensities can be achieved, or where long-term effects are desired. Our work also emphasizes that *C*. *elegans* may serve as an additional testbed for novel rhodopsin optogenetic tools.

## Supporting information

S1 Fig ChR2 variants expressed in body-wall muscle cells. Expression of ChR2(L132C)::YFP, ChR2(C128S;L132C;H134R)::YFP, and ChR2(L132C;H134R;T159C). Arrowheads indicate muscle arms which body-wall muscle cells extend towards the neuronal processes to form neuromuscular junctions. Scale bar is 10 μm.
(JPG)

S2 Fig Description of behavioral assays in *C*. *elegans* expressing de- or hyperpolarizing rhodopsin-based tools in body-wall muscle cells. Photostimulation leads to either contraction or elongation of the body. Changes in body length are analyzed in videos of free moving animals (for further details see Materials and methods).
(JPG)

S3 Fig ChR2 variants expressed in body-wall muscle cells induce body contractions depending on the light intensity used. Dependence of body contractions on light intensity in animals expressing **(a)** ChR2(H134R)::YFP, **(b)** ChR2(C128S)::YFP, **(c)** ChR2(L132C)::YFP, **(d)** ChR2(C128S;H134R)::YFP, **(e)** ChR2(H134R;D156C)::YFP, **(f)** ChR2(C128S;L132C;H134R)::YFP, **(g)** ChR2(L132C;H134R;T159C), and **(h)** Quint::YFP in body-wall muscle cells of *C*. *elegans*. Reductions in body length were recorded in response to light stimuli (1, 2, or 5 s, 450–490 nm) of intensities in the range of 0.03 μW/mm^2^ to 2.41 mW/mm^2^. Shown is the mean normalized body length (± SEM) calculated relative to the initial length of the animal; n = number of animals.
(JPG)

S4 Fig Quint induces body contractions over a broad wavelength spectrum. **(a)** Dependence of body contractions on wavelength (430–620 nm) in animals expressing Quint::YFP in body-wall muscle cells of *C*. *elegans*. Reductions in body length were recorded in response to light stimuli (5 s; 300 μW/mm^2^). Shown is the mean normalized body length (± SEM) calculated relative to the initial length of the animal. **(b)** Change in body length of animals expressing Quint::YFP (430–620 nm) or ChR2(H134R)::YFP (580 and 620 nm) following photostimulation (5 s; 300 μW/mm^2^) at different wavelengths. **(c)** Body contractions of animals expressing Quint::YFP in response to prolonged stimulation with red light (15 s; 620 nm; 300 μW/mm^2^). Shown is the mean normalized body length (± SEM) calculated relative to the initial length of the animal; n = number of animals. **p<0.01, ***p<0.001.
(JPG)

S5 Fig ACR variants induce body elongations depending on the used light intensity. Expression of ACR1::eYFP and ACR2::eYFP in cholinergic neurons of *C*. *elegans*. Scale bar is 50 μm. **b)** Body length calculated of animals expressing ACR1 or ACR2 expressed in cholinergic neurons during and after a 5 s light stimulus (1 mW/mm^2^; 470 nm). Shown is the mean normalized body length (± SEM) relative to the initial length of the animal. **(c)** Maximal changes in body length induced by the tested hyperpolarizers. Shown is the mean normalized change in body length (± SEM) relative to the initial length of the animal. **(d)** Light wavelength and intensity dependence of the body elongation of transgenic animals expressing ACR1 or ACR2 in cholinergic neurons. Dependence of body elongations on light intensity in animals expressing ACR1 (cholinergic neuron) **(e)**, ACR2 (cholinergic neuron) **(f)**, ZipACR (BWM) **(g)** or ACR1(C102A) (BWM) **(h)**. Elongations in body length were recorded in response to light stimuli (2 or 5 s, 470 or 520 nm) of intensities in the range of 0.5 μW/mm^2^ to 1 mW/mm^2^. Shown is the mean normalized body length (± SEM) calculated relative to the initial length of the animal. **(i)** Partial closing of ACR1(C102A) channel with red light (620–660 nm). The photocurrents were recorded from a HEK293 cell held at -40 mV at the amplifier output. The duration of the activating 515-nm light pulse was 10 ms (for further details see Methods section). **(j)** Body length in response to repetitive photostimulation (5 s, 5 s ISI, 80 μW/mm^2^, 470 or 520 nm) of ACR1 or ACR2 **(k)** in animals expressing the respective channel in body-wall muscles or cholinergic neurons. Blue and green bars indicate illumination period. n = number of animals. Significance is given relative to body length before illumination: *p<0.05, **p<0.01, ***p<0.001. For easier comparison of effects in BWMs and cholinergic neurons results from BWMs presented in Fig 3g, 3i and 3j have been included in S5c,j,k Fig.
(JPG)

S6 Fig Membrane potential shift following ACR-mediated hyperpolarization may indicate action of Cl^-^ efflux pumps. Shown are the mean voltage traces of four experiments each, filtered as a sliding average (2000 frame window at 5 kHz sampling). Two consecutive dark and light stimulation periods from Fig 5c are shown (ACR1 and ACR2, as indicated). The upward (depolarizing) trend in the baseline is visualized by graphical regression.
(JPG)

S1 Video A representative video obtained from animals expressing ChR2(C128S;L132C;H134R) in body-wall muscle, before and during the light stimulus phase. A body contraction is seen when the stimulation light is turned on.
(MP4)

S2 Video A representative video obtained from animals expressing ACR1(C102) in body-wall muscle, before and during the light stimulus phase. A body elongation and reduction in locomotion is seen when the stimulation light is turned on.
(MP4)

S3 Video A representative video obtained from animals expressing ACR1 in cholinergic neurons, before and during the light stimulus phase. A body elongation and stop of locomotion is seen when the stimulation light is turned on.
(MP4)

S1 Data This file contains raw data of contractions assays related to depolarizers presented in this paper. (XLSX)

S2 Data This file contains raw data of contractions assays and electrophysiological recordings related to hyperpolarizers presented in this paper. (XLSX)

S3 Data This file contains plasmid maps of transgenic animals presented in this paper. (DOCX)

S4 Data This file contains the plasmid map of pmyo-3::ChR2(H134R). (GBK)

S5 Data This file contains the plasmid map of pmyo-3::ChR2(C128S). (GBK)

S6 Data This file contains the plasmid map of pmyo-3::ChR2(L132C). (GBK)

S7 Data This file contains the plasmid map of pmyo-3::ChR2(T159C). (GBK)

S8 Data This file contains the plasmid map of pmyo-3::ChR2(C128S;H134R). (GBK)

S9 Data This file contains the plasmid map of pmyo-3::ChR2(H134R;D156C). (GBK)

S10 Data This file contains the plasmid map of pmyo-3::ChR2(T159C). (GBK)

S11 Data This file contains the plasmid map of pmyo-3::ChR2(C128S;L132C;H134R). (GBK)

S12 Data This file contains the plasmid map of pmyo-3::ChR2(L132C;H134R;T159C). (GBK)

S13 Data This file contains the plasmid map of pmyo-3::Quint. (GBK)

S14 Data This file contains the plasmid map of pmyo-3::ACR1. (GBK)

S15 Data This file contains the plasmid map of punc-17::ACR1. (GBK)

S16 Data This file contains the plasmid map of pmyo-3::ACR2. (GBK)

S17 Data This file contains the plasmid map of punc-17::ACR2. (GBK)

S18 Data This file contains the plasmid map of pmyo-3::ACR1(C102A). (GBK)

S19 Data This file contains the plasmid map of pmyo-3::ZipACR. (GBK)

S20 Data This file contains the plasmid map of pmyo-3::NpHR. (GBK)
