# Supplementary figures and images for: Rhodopsin optogenetic toolbox v2.0 for light-sensitive excitation and inhibition in Caenorhabditis elegans
Source: PLoS One. 2018 Feb 1;13(2):e0191802. doi: 10.1371/journal.pone.0191802 (PMC5794093; doi:10.1371/journal.pone.0191802)

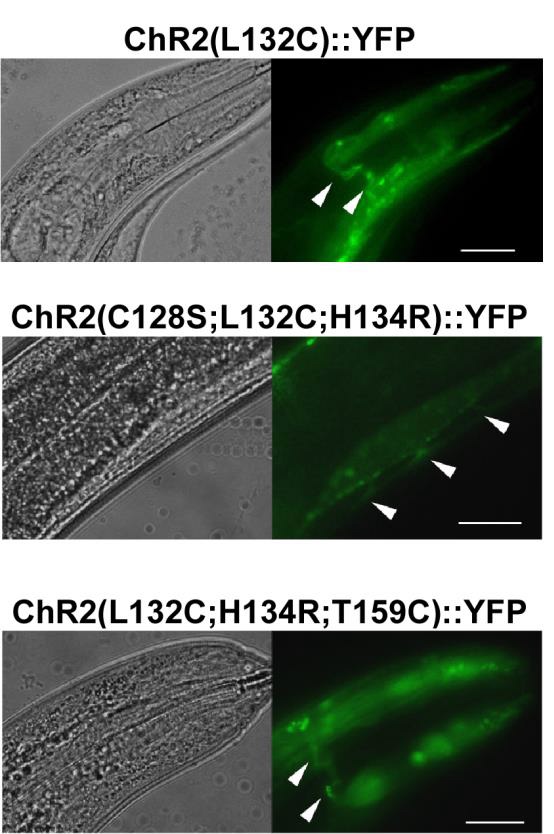

Supplement: S1 Fig — Expression of ChR2(L132C)::YFP, ChR2(C128S;L132C;H134R)::YFP, and ChR2(L132C;H134R;T159C). Arrowheads indicate muscle arms which body-wall muscle cells extend towards the neuronal processes to form neuromuscular junctions. Scale bar is 10 μm. (JPG) [file pone.0191802.s001.jpg]

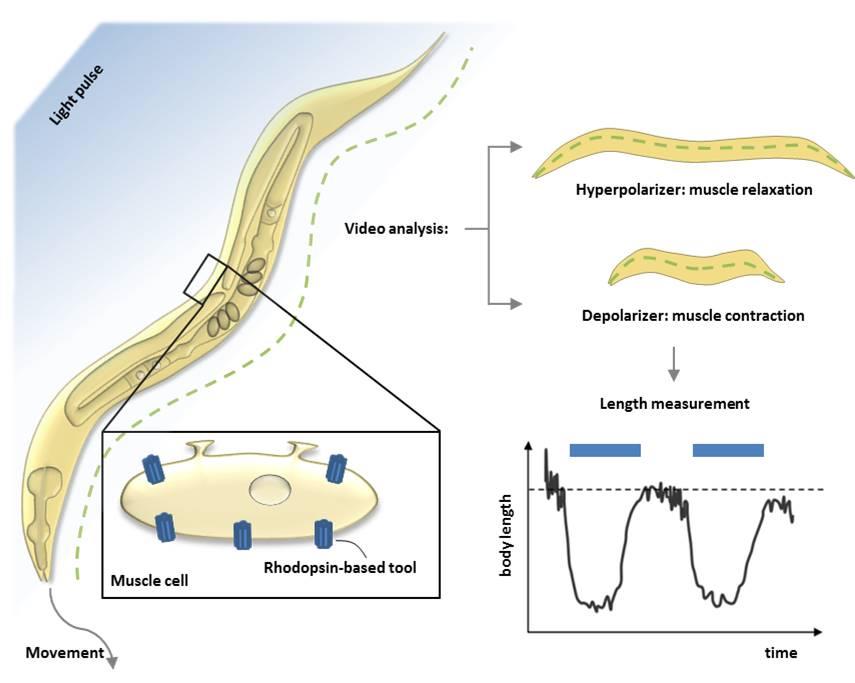

Supplement: S2 Fig — Photostimulation leads to either contraction or elongation of the body. Changes in body length are analyzed in videos of free moving animals (for further details see Materials and methods). (JPG) [file pone.0191802.s002.jpg]

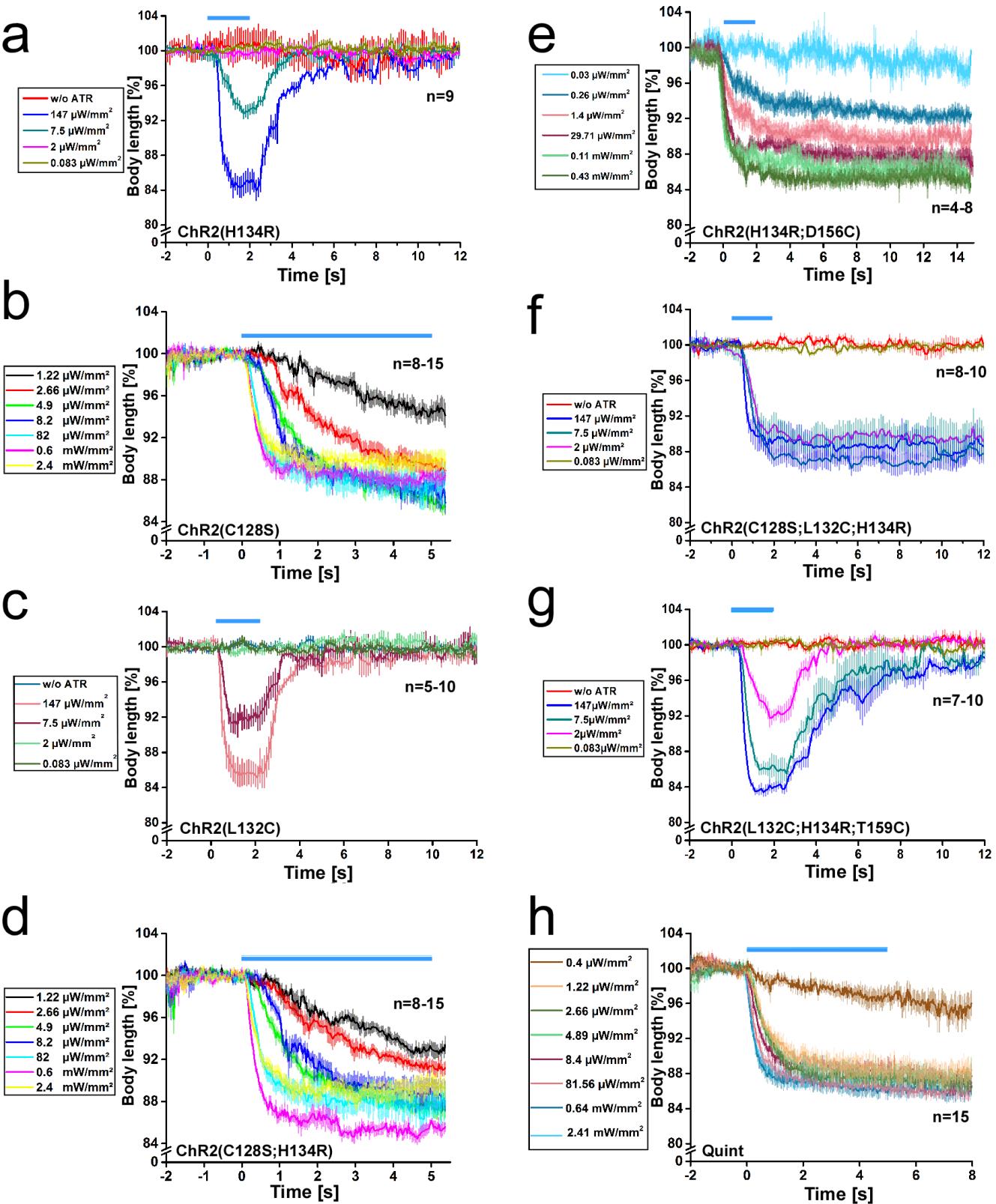

Supplement: S3 Fig — Dependence of body contractions on light intensity in animals expressing (a) ChR2(H134R)::YFP, (b) ChR2(C128S)::YFP, (c) ChR2(L132C)::YFP, (d) ChR2(C128S;H134R)::YFP, (e) ChR2(H134R;D156C)::YFP, (f) ChR2(C128S;L132C;H134R)::YFP, (g) ChR2(L132C;H134R;T159C), and (h) Quint::YFP in body-wall muscle cells of C. elegans. Reductions in body length were recorded in response to light stimuli (1, 2, or 5 s, 450–490 nm) of intensities in the range of 0.03 μW/mm2 to 2.41 mW/mm2. Shown is the mean normalized body length (± SEM) calculated relative to the initial length of the animal; n = number of animals. (JPG) [file pone.0191802.s003.jpg]

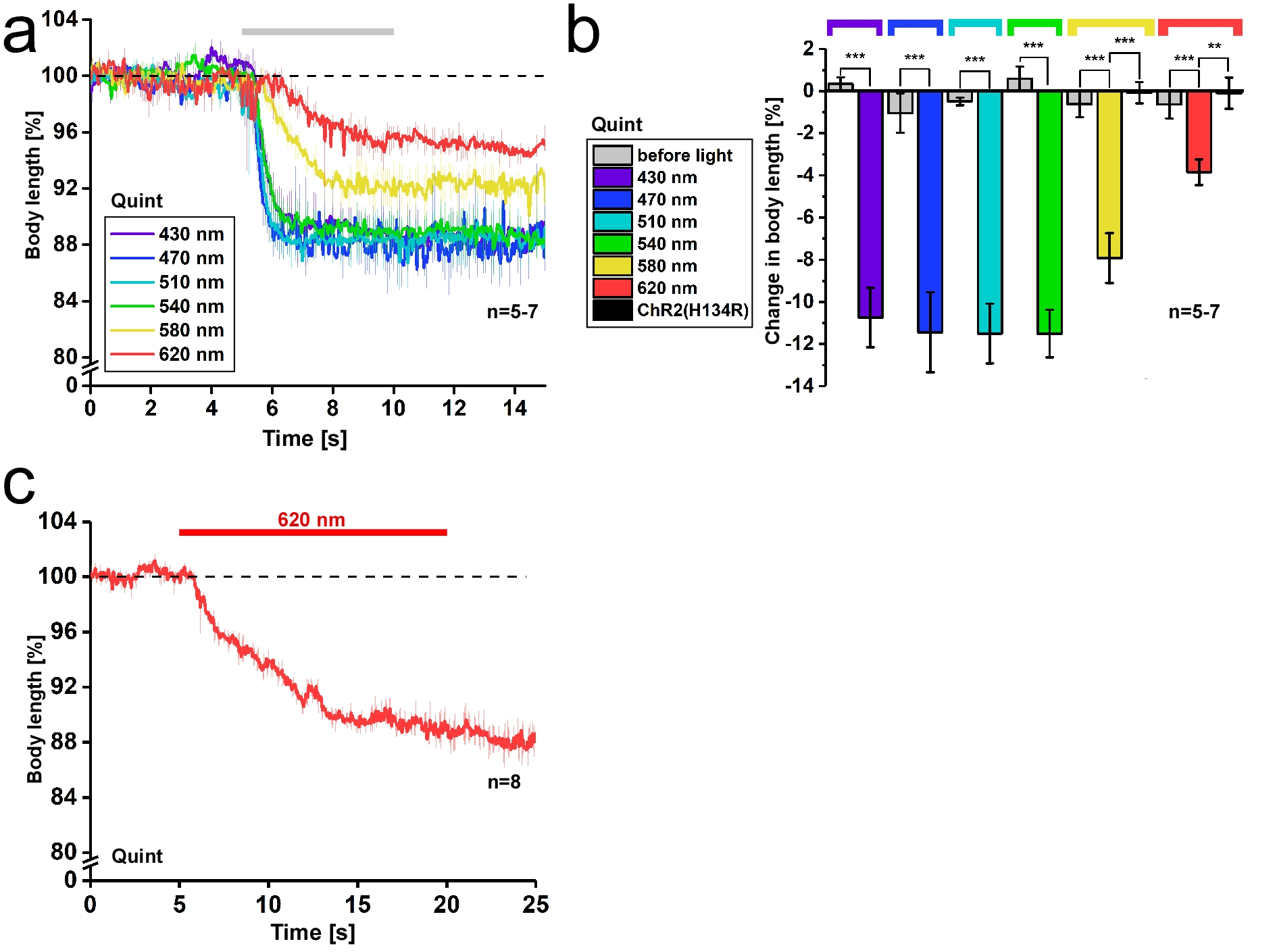

Supplement: S4 Fig — (a) Dependence of body contractions on wavelength (430–620 nm) in animals expressing Quint::YFP in body-wall muscle cells of C. elegans. Reductions in body length were recorded in response to light stimuli (5 s; 300 μW/mm2). Shown is the mean normalized body length (± SEM) calculated relative to the initial length of the animal. (b) Change in body length of animals expressing Quint::YFP (430–620 nm) or ChR2(H134R)::YFP (580 and 620 nm) following photostimulation (5 s; 300 μW/mm2) at different wavelengths. (c) Body contractions of animals expressing Quint::YFP in response to prolonged stimulation with red light (15 s; 620 nm; 300 μW/mm2). Shown is the mean normalized body length (± SEM) calculated relative to the initial length of the animal; n = number of animals. **p<0.01, ***p<0.001. (JPG) [file pone.0191802.s004.jpg]

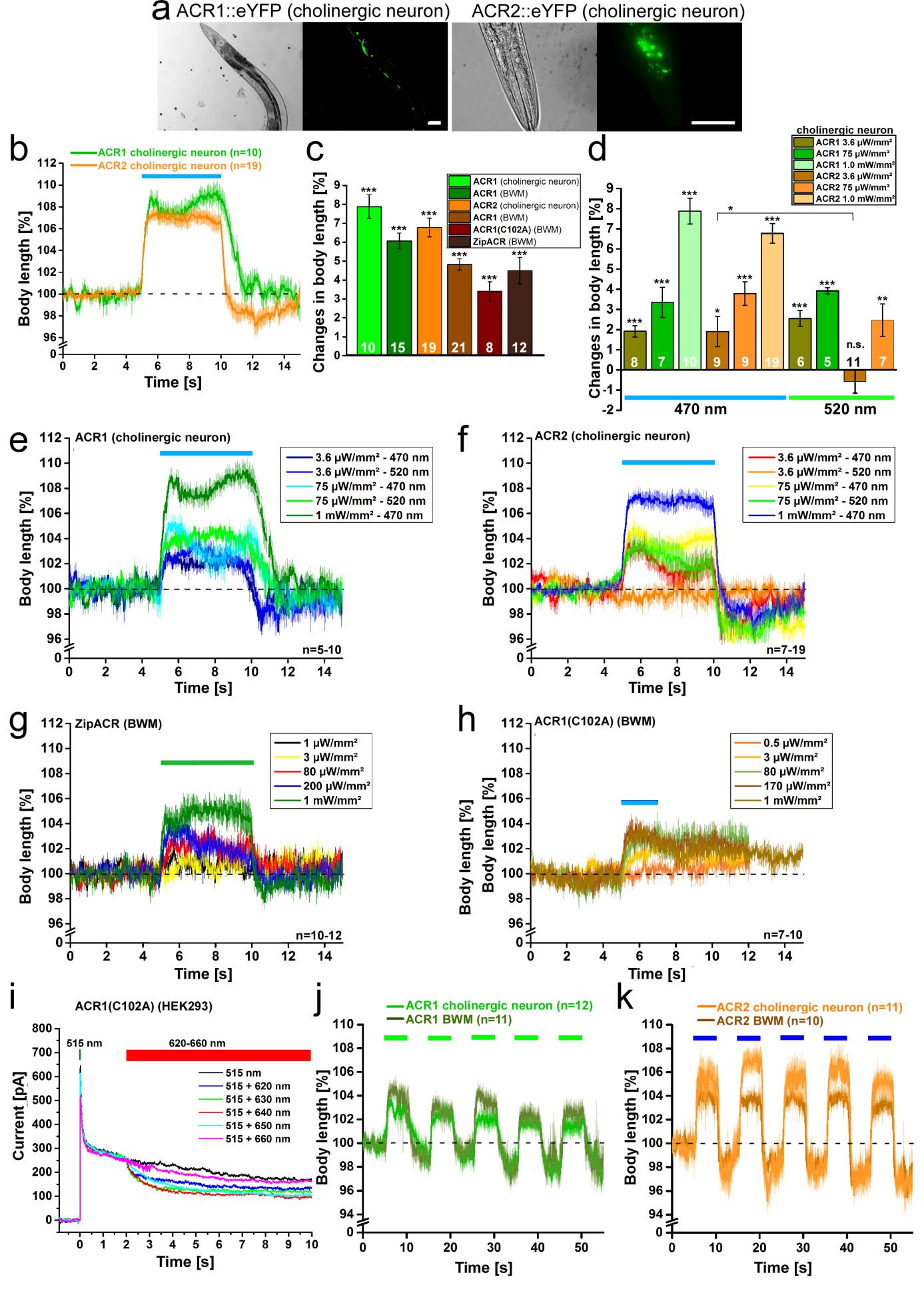

Supplement: S5 Fig — Expression of ACR1::eYFP and ACR2::eYFP in cholinergic neurons of C. elegans. Scale bar is 50 μm. b) Body length calculated of animals expressing ACR1 or ACR2 expressed in cholinergic neurons during and after a 5 s light stimulus (1 mW/mm2; 470 nm). Shown is the mean normalized body length (± SEM) relative to the initial length of the animal. (c) Maximal changes in body length induced by the tested hyperpolarizers. Shown is the mean normalized change in body length (± SEM) relative to the initial length of the animal. (d) Light wavelength and intensity dependence of the body elongation of transgenic animals expressing ACR1 or ACR2 in cholinergic neurons. Dependence of body elongations on light intensity in animals expressing ACR1 (cholinergic neuron) (e), ACR2 (cholinergic neuron) (f), ZipACR (BWM) (g) or ACR1(C102A) (BWM) (h). Elongations in body length were recorded in response to light stimuli (2 or 5 s, 470 or 520 nm) of intensities in the range of 0.5 μW/mm2 to 1 mW/mm2. Shown is the mean normalized body length (± SEM) calculated relative to the initial length of the animal. (i) Partial closing of ACR1(C102A) channel with red light (620–660 nm). The photocurrents were recorded from a HEK293 cell held at -40 mV at the amplifier output. The duration of the activating 515-nm light pulse was 10 ms (for further details see Methods section). (j) Body length in response to repetitive photostimulation (5 s, 5 s ISI, 80 μW/mm2, 470 or 520 nm) of ACR1 or ACR2 (k) in animals expressing the respective channel in body-wall muscles or cholinergic neurons. Blue and green bars indicate illumination period. n = number of animals. Significance is given relative to body length before illumination: *p<0.05, **p<0.01, ***p<0.001. For easier comparison of effects in BWMs and cholinergic neurons results from BWMs presented in Fig 3g, 3i and 3j have been included in S5c,j,k Fig. (JPG) [file pone.0191802.s005.jpg]

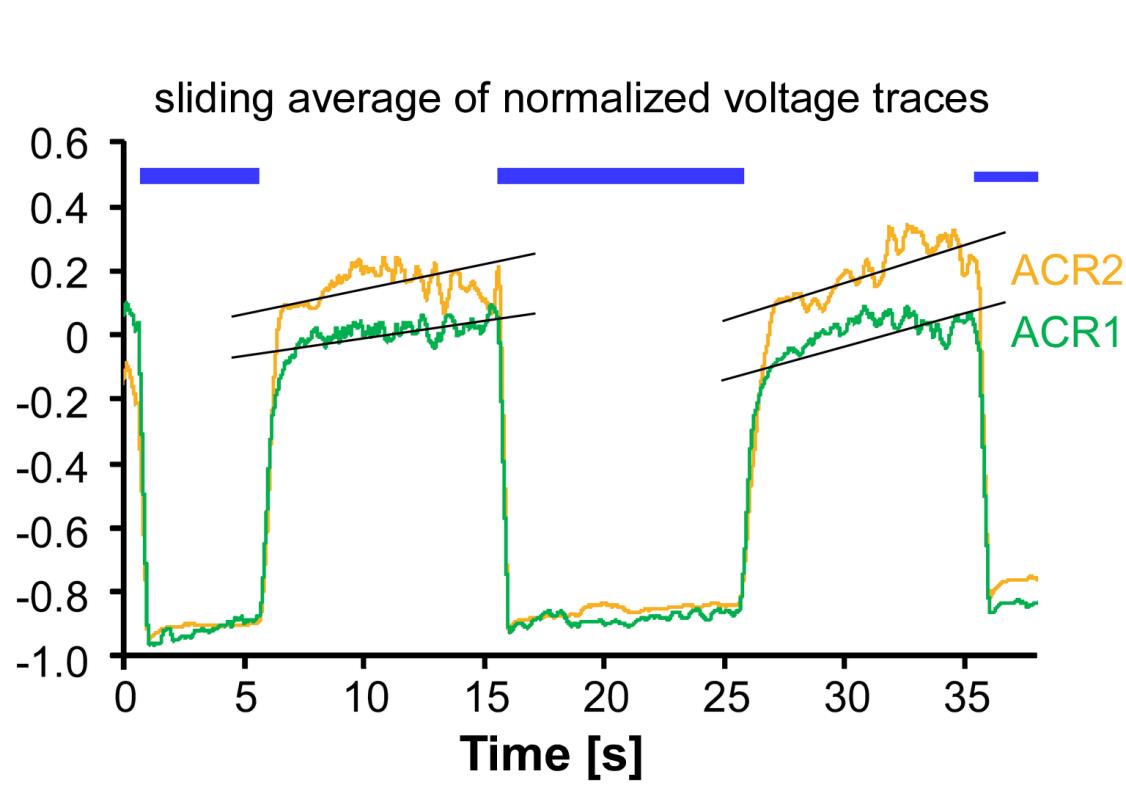

Supplement: S6 Fig — Shown are the mean voltage traces of four experiments each, filtered as a sliding average (2000 frame window at 5 kHz sampling). Two consecutive dark and light stimulation periods from Fig 5c are shown (ACR1 and ACR2, as indicated). The upward (depolarizing) trend in the baseline is visualized by graphical regression. (JPG) [file pone.0191802.s006.jpg]
