# Supplementary material for: Rhodopsin optogenetic toolbox v2.0 for light-sensitive excitation and inhibition in Caenorhabditis elegans
Source: PLoS One. 2018 Feb 1;13(2):e0191802. doi: 10.1371/journal.pone.0191802 (PMC5794093; doi:10.1371/journal.pone.0191802)
Supplement: S3 Data — (DOCX) [file pone.0191802.s012.docx]

**ZX299**: *lin-15(n765ts-);zxEx22[pmyo-3::ChR2(H134R)::YFP;lin-15+]*****

**ZX444:** *lin-15(n765ts-);zxEx301[pmyo-3::NpHR::ecfp;lin-15+]*

**ZX838**: *lin-15(n765ts-);zxEx423[pmyo-3::ChR2(C128S)::YFP;lin-15+]*

**ZX954**: *lin-15(n765ts-);zxEx468[pmyo-3::ChR2(C128S;H134R)::YFP;lin-15+]*

**ZX1295**: *lin-15(n765ts-);zxEx477[pmyo-3::ChR2(C128S;H134R)::YFP(100ng/µl);lin-15+]*

**ZX1296**: *lin-15(n765ts-); zxEx478[pmyo-3::ChR2(C128S;H134R)::YFP(40ng/µl);lin-15+]*

**ZX1297**: *lin-15(n765ts-);zxEx479[pmyo-3::ChR2(C128S;H134R)::YFP(10ng/µl);lin-15+]*

**ZX1298**: *lin-15(n765ts-);zxEx480[pmyo-3::ChR2(C128S;H134R)::YFP(2ng/µl);lin-15+]*

**ZX1166**: N2;*zxEx536[pmyo-3::ChR2(T159C)::YFP;pmyo-2::mCherry]*

**ZX1167**: N2;*zxEx537[pmyo-3::ChR2(H134R;T159C)::YFP;pmyo-2::mCherry]*

**ZX1299**: *lin-15(n765ts-);zxEx477[pmyo-3::ChR2-Quint::YFP(100ng/µl);lin-15+]*

**ZX1300**: *lin-15(n765ts-);zxEx478[pmyo-3::ChR2-Quint::YFP(40ng/µl); lin-15+]*

**ZX1301**: *lin-15(n765ts-);zxEx479[pmyo-3::ChR2-Quint::YFP (10ng/µl);lin-15+]*

**ZX1302**: *lin-15(n765ts-);zxEx480[pmyo-3::ChR2-Quint::YFP(2ng/µl);lin-15+]*

**

**ZX1788**: N2;*zxEx1036[pmyo-3::ChR2(H134R;D156C)::YFP(30ng/µl);pELT-2::mCherry]*

**ZX1826**: N2;*zxEx740[pmyo-3::ChR2(C128S;L132C;H134R)::YFP;pmyo-2::mCherry]*

**

**ZX1827**: N2;*zxEx741[pmyo-3::ChR2(L132C)::YFP(80ng/µl);pmyo-2::mCherry]*

**

**ZX1830**: N2;*zxEx744[pmyo-3::ChR2(L132C;H134R;T159C)::YFP(80ng/µl);pmyo-2::mCherry]*

**

**ZX2022**: N2;*zxEx1031[pmyo-3::ACR1::eYFP(30ng/µl);pmyo-2::mCherry]*

**

**ZX2023**: N2;*zxEx1032[pmyo-3::ACR2::eYFP(30ng/µl);pmyo-2::mCherry]*

**ZX2024**: N2;*zxEx1033[punc-17::ACR1::eYFP(30ng/µl); pmyo-3::mCherry]*

**ZX2026**: N2;*zxEx1034[punc-17::ACR2::eYFP(30ng/µl);pmyo-3::mCherry]*

**ZX2206**: *zxEx1073[pmyo-3::ACR1(C102A)::eYFP;pmyo-2::mCherry] in N2 (30ng/µl)*

**ZX2207**: *zxEx1074[pmyo-3::ZipACR::eYFP;pmyo-2::mCherry] in N2 (30ng/µl)*
